# Inhibition of retinoic acid signaling in proximal tubular epithelial cells protects against acute kidney injury

**DOI:** 10.1172/jci.insight.173144

**Published:** 2023-10-23

**Authors:** Min Yang, Lauren N. Lopez, Maya Brewer, Rachel Delgado, Anna Menshikh, Kelly Clouthier, Yuantee Zhu, Thitinee Vanichapol, Haichun Yang, Raymond C. Harris, Leslie Gewin, Craig R. Brooks, Alan J. Davidson, Mark de Caestecker

**Affiliations:** 1 Department of Medicine, Division of Nephrology, Vanderbilt University Medical Center, Nashville, Tennessee, USA.; 2 Department of Anesthesiology and Critical Care, University of Pennsylvania, Philadelphia, Pennsylvania, USA.; 3 Department of Molecular Medicine & Pathology, The University of Auckland, Auckland, New Zealand.; 4 Department of Pathology, Vanderbilt University Medical Center, Nashville, Tennessee, USA.; 5 Washington University in St. Louis School of Medicine and the St. Louis Veterans Affairs Hospital, St. Louis, Missouri, USA.

**Keywords:** Nephrology, Molecular pathology

## Abstract

Retinoic acid receptor (RAR) signaling is essential for mammalian kidney development but, in the adult kidney, is restricted to occasional collecting duct epithelial cells. We now show that there is widespread reactivation of RAR signaling in proximal tubular epithelial cells (PTECs) in human sepsis-associated acute kidney injury (AKI) and in mouse models of AKI. Genetic inhibition of RAR signaling in PTECs protected against experimental AKI but was unexpectedly associated with increased expression of the PTEC injury marker Kim1. However, the protective effects of inhibiting PTEC RAR signaling were associated with increased Kim1-dependent apoptotic cell clearance, or efferocytosis, and this was associated with dedifferentiation, proliferation, and metabolic reprogramming of PTECs. These data demonstrate the functional role that reactivation of RAR signaling plays in regulating PTEC differentiation and function in human and experimental AKI.

## Graphical abstract

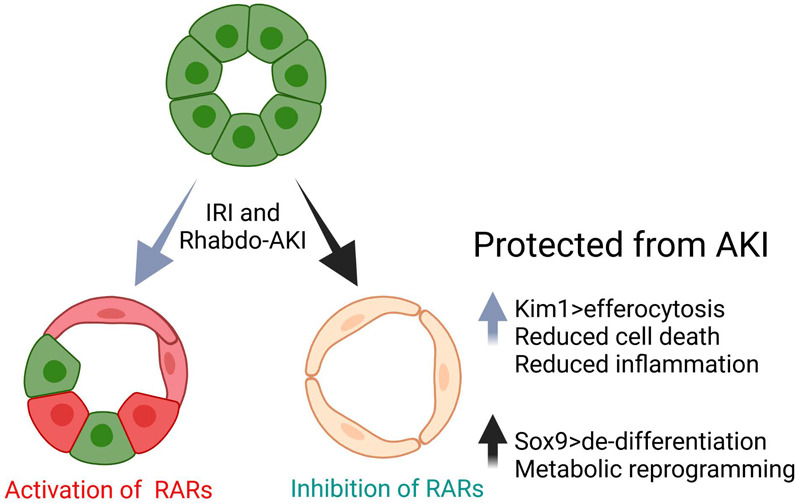

## Introduction

Acute kidney injury (AKI), which occurs in 10%–20% of adults hospitalized in developed countries with acute illnesses (1), is an independent predictor of mortality (2) and increases the risk of chronic kidney disease (CDK) (3, 4). Current treatment options for AKI are limited, and no therapeutic interventions have been shown to improve outcomes after AKI (5, 6). In part, this is because AKI is a heterogeneous condition that is defined clinically based on changes in renal function that do not reflect the diverse pathophysiological responses (7). The most common form of AKI is acute tubular injury (ATI), caused by nephrotoxic drugs, reduced renal perfusion, muscle injury leading to Rhabdomyolysis-AKI (Rhabdo-AKI), trauma and surgery, and sepsis-associated AKI (SA-AKI) (7, 8). Molecular analyses of human kidneys from patients with AKI have begun to elucidate pathophysiological mechanisms (9–11), but limited access to tissues continues to hamper our ability to identify molecular mechanisms of AKI (12). An experimental model that has been used extensively to evaluate the cellular responses over time is the rodent ischemia reperfusion–induced model of AKI (IRI-AKI) (13). This induces defined injury with dominant damage to proximal tubular epithelial cells (PTECs), the most abundant and metabolically active cells in the kidney (14). Other models used to evaluate AKI mechanisms include cisplatin-AKI (CP-AKI), modeling the effects of chemotherapy in patients with solid organ malignancies (15), and Rhabdo-AKI, modeling the effects of crush injuries (16), heme-induced AKI in sepsis, sickle cell disease, and cardiac surgery (17, 18).

KIM1 (encoded by *HAVCR1*) is a phosphatidylserine (PS) receptor first described as a marker of injury in dedifferentiated PTECs after IRI-AKI (19). Kim1 is upregulated in PTECs in a variety of rodent models and in patients with AKI (20–23). Kim1 triggers efferocytosis of apoptotic cells by PTECs (24, 25), while loss-of-function studies using *Havcr1* mutant mice show that loss of Kim1 expression or PS binding function exacerbates ATI, increases tubular cell apoptosis and necrosis, and increases inflammation after AKI (26–28). Decreased inflammation is mediated by decreasing proinflammatory secondary necrosis of apoptotic cells that have not undergone clearance (29), and suppression of inflammatory responses by PTECs that have phagocytosed apoptotic cells (27). However, in other settings, overexpression of Kim1 may provoke inflammation and fibrosis. For example, a conditional mouse mutant that induces persistent Kim1 expression in renal epithelium provokes renal inflammation and fibrosis in adult mice (30). In addition, persistent Kim1 expression is a feature of inflammatory PTECs that have failed to undergo productive repair after AKI (10, 31, 32). On this basis, it is unclear whether enhancing Kim1 expression after AKI would be protective or would cause more severe injury and CKD.

The retinoic acid (RA) signaling plays an essential role in mammalian kidney development (33), but in the adult kidney, RA signaling is restricted to occasional cells within the collecting duct (CD) system (34). RA metabolites are generated in a 2-step process that includes the rate-limiting oxidation of retinaldehyde to RA by retinaldehyde dehydrogenases (RALDHs, encoded by the *ALDH1A* gene family). Once generated, RAs may act in an autocrine or paracrine fashion and mediate effects by binding to the RA receptors (RARs), which activate or repress transcriptional targets to regulate diverse cellular functions. These effects are cell type and context dependent. For example, RA can promote epithelial differentiation in some circumstances, while in others, it enhances stem cell–like properties (35–37), and RAR signaling promotes heart, limb, and fin appendage regeneration in amphibia and fish (38–40).

We have shown that Raldh2 and Raldh3 are induced in peritubular monocyte/macrophages and myofibroblasts after IRI-AKI and have used RAR reporter mice to show that RAR signaling is activated in adjacent renal macrophages and in injured Kim1^+^ PTECs after IRI-AKI (41). Systemic inhibition of RAR signaling worsens renal injury and increases inflammatory macrophage activation after IRI-AKI, and macrophage-depletion studies show that worsening injury after inhibition of RAR signaling is dependent on macrophages (41). These findings are consistent with the known antiinflammatory effects of RAR signaling in macrophages (42–46) and the protective effects of exogenous retinoids on renal injury in toxin and IRI-AKI models (41, 47, 48), in which RA treatments reduce both tubular injury and inflammation in the kidney (41, 47). However, these findings do not explain the functional role that activation of RAR signaling in PTECs plays in the cellular responses to injury, nor whether this response to injury is conserved in patients or in other experimental models of AKI.

We now show that PTEC RAR signaling is activated in patients with SA-AKI and mice with Rhabdo-AKI, and we show that inhibition of RAR signaling in PTECs protects against Rhabdo-AKI and, to a lesser extent, IRI-AKI. This is associated with increased Kim1-dependent efferocytosis by PTECs. Increased Kim1 expression is not associated with increased tubular injury but is associated with enhanced dedifferentiation, proliferation, and metabolic reprogramming of PTECs. These findings provide the first gain-of-function evidence to our knowledge that enhanced Kim1 expression by injured PTECs may play a protective role in AKI and suggest that activation of RAR signaling after AKI is a compensatory response that maintains the mature differentiation state and quiescence of surviving PTECs after AKI.

## Results

### RA signaling is activated in PTECs in human and experimental AKI.

**Figure 1:**
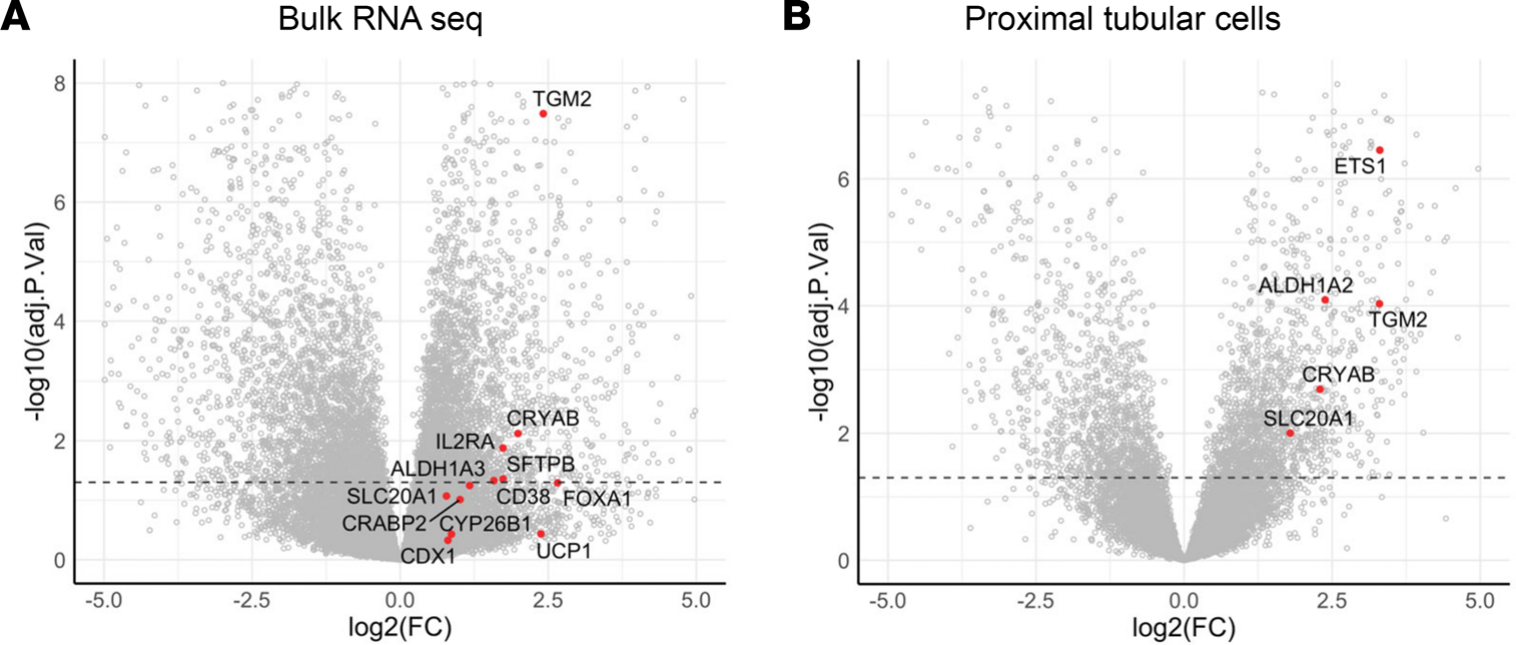
Activation of retinoic acid receptor (RAR) signaling in patients with sepsis-associated AKI. Gene set enrichment analysis (GSEA) of bulk RNA as well as cell-specific snRNA-Seq kidney data sets from patients with SA-AKI compared with age-matched controls from Hinze et al. (11). (**A** and **B**) Volcano plots indicating fold change in expression of core enrichment genes (AKI versus controls) from bulk RNA and PTEC snRNA-Seq data sets. Dotted line indicates *P* < 0.05.

To determine whether RAR signaling is activated in human AKI, we evaluated bulk RNA-Seq and single nuclear RNA-Seq (snRNA-Seq) data obtained from a validated cohort of samples from patients with severe SA-AKI (see Supplemental Methods for a summary of the clinical and validation criteria used for these studies; supplemental material available online with this article; https://doi.org/10.1172/jci.insight.173144DS1) (49). Gene set enrichment analysis (GSEA) was performed to assess enrichment with 27 validated RAR target genes (26 upregulated and 1 variably regulated by RA in different cell types) (50) and 3 RA regulatory genes, *ALDH1A2*, *ALDH1A3*, and *CYP26B1*, which are upregulated in mouse kidneys after IRI-AKI (Supplemental Table 1) (41). There was enrichment for RAR target genes in bulk and PTEC RNA-Seq data sets (Table 1; Figure 1, A and B; and Supplemental Table 2), while thick ascending limb (TAL) and renal leukocyte populations showed less robust enrichment for RAR target genes (Table 1 and Supplemental Table 2). There was no significant enrichment for RAR target genes in CD or other snRNA-Seq cell populations in this data set (Table 1 and Supplemental Table 2). More detailed analysis of PTEC subsets indicates that RAR target genes are predominantly activated in injured, repairing, and failed repair PTECs (which also express KIM1) but also in differentiated S3 segment PTECs, which are found in the outer stripe of the outer medulla, in AKI samples (Supplemental Figure 1). RAR target genes are also expressed in “new” injured and repairing TAL cells (Supplemental Figure 2) and in monocyte/macrophage lineages (Supplemental Figure 3).

**Figure 2:**
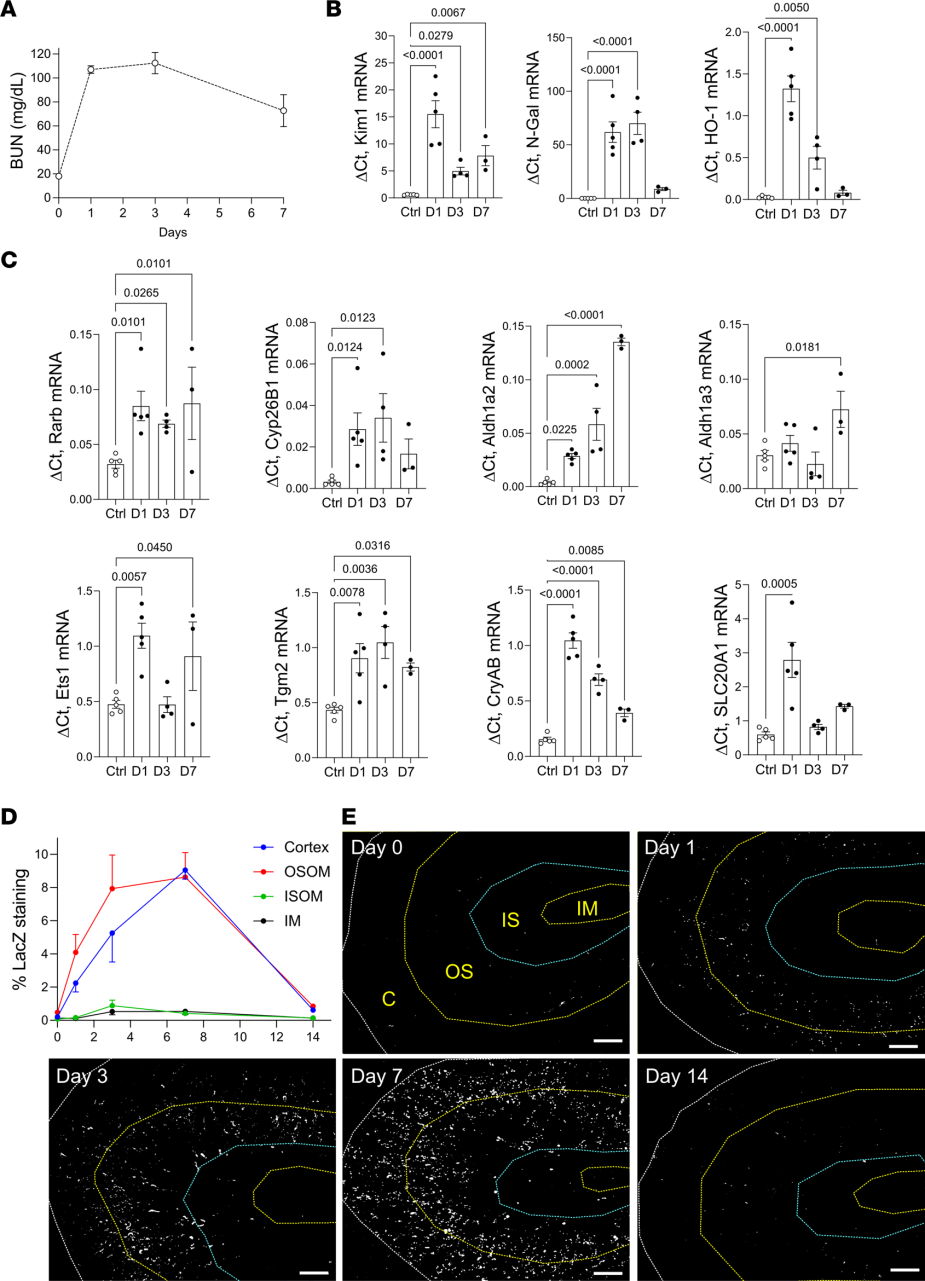
Widespread activation of RAR signaling after Rhabdo-AKI. (**A**) BUN time course after Rhabdo-AKI in BALB/c mice: 15 mice before injury (day 0) and day 1 after injury, 9 at day 3, and 3 at day 7. (**B**) Expression of injury marker mRNAs. (**C**) RAR target gene mRNAs in 5 uninjured and day 1 mice, 4 at day 3, and 3 at day 7. (**D**) Spatial distribution and kinetics of RAR signaling after Rhabdo-AKI in RARE-LacZ mice. Kidneys stained for LacZ activity, 3 before injury (day 0); 4 at days 1 and 3; 3 at day 7; and 5 at day 14 after injury. (**E**) Percentage of area staining for LacZ in the cortex (C); OSOM; inner stripe of the outer medulla (ISOM); and inner medulla (IM). LacZ staining at different time points after injury. LacZ staining is pseudocolored in white, and kidney regions are demarcated by dotted lines in the first panel. Scale bars: 500 mM. **B** and **C** used 1-way ANOVA; if *P* < 0.05, *q* values are shown for between-group comparisons corrected for repeat testing.

These data are consistent with our prior findings using the *RARE-hsp68-LacZ* (RARE-LacZ) transgenic reporter mouse line, which expresses bacterial LacZ/β*-*Galactosidase (β-Gal) in cells upon activation of canonical RAR signaling (33, 34, 51). There was strong activation of the reporter in Kim1^+^ PTECs and F480^+^ renal macrophages, with less activation of the reporter in THP1^+^ TAL and Dolichos biflorus^+^ (DB^+^) CD cells after IRI-AKI (41). To determine whether there were differences in the distribution of RAR activation in different models of AKI, we evaluated RAR activation in a mouse model of Rhabdo-AKI induced by intramuscular injection of glycerol (52). In this model, there is upregulation of PTEC and distal tubular injury markers, Kim1 and N-Gal/Lcn2 mRNAs, respectively, and upregulation of the early injury response gene, *Heme Oxygenase 1 (Ho1)* at 24–72 hours after AKI (Figure 2, A and B). This is associated with upregulation of the RAR target gene, *Rarb*, and other RAR targets and regulators that were upregulated in the bulk RNA-Seq and PTEC snRNA-Seq human SA-AKI data sets (Figure 2C). To evaluate the kinetics and cellular distribution of RAR signaling, we evaluated the distribution of the RARE-LacZ reporter after Rhabdo-AKI. There was widespread activation of the reporter throughout the cortex and outer stripe of the outer medulla (OSOM) of the kidney, peaking 3–7 days after injury and returning to baseline expression by day 14 (Figure 2, D and E). This contrasts with IRI-AKI in which RARE-LacZ reporter activation is largely restricted to the OSOM, and is more transient, peaking 12–24 hours after injury and returning to baseline levels by day 7 (41).

**Figure 3:**
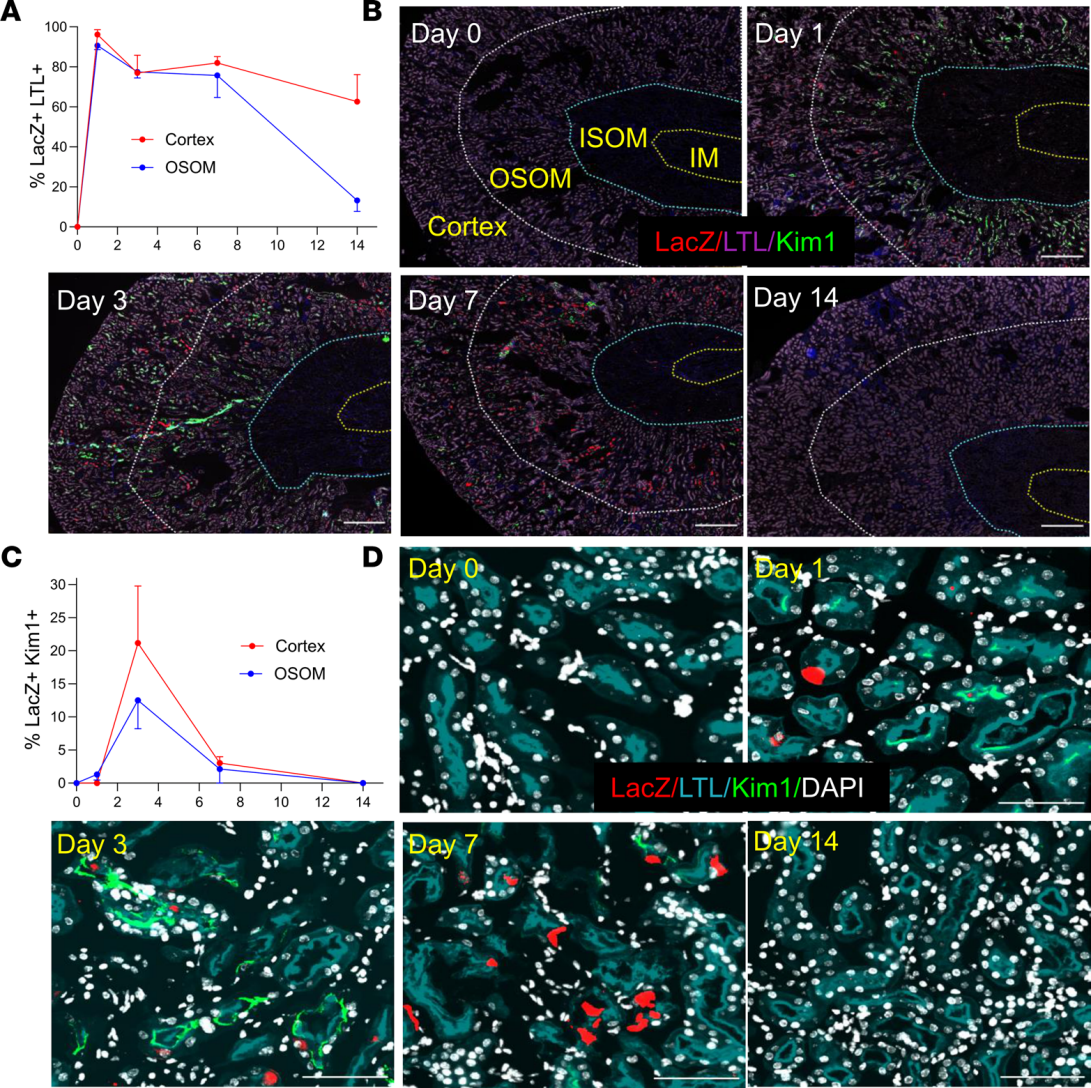
RAR signaling is extensively activated in Kim1^–^ PTECs after Rhabdo-AKI. RARE-LacZ reporter mice were used to evaluate the cellular localization of RAR signaling after Rhabdo-AKI. (**A** and **C**) Percentage of LacZ^+^ cells in LTL^+^ and Kim1^–^ PTECs (**A**) and in Kim1^+^ PTECs (**C**) at the different time points after injury in the cortex and OSOM: 2 mice before injury (day 0) and 4 at days 1 and 3, 3 at day 7, and 5 at day 14 after injury. (**B** and **D**) Images showing LacZ, LTL, and Kim1 staining in the cortex and OSOM. (**B**) Low-power images of the kidneys; regions of the kidney are demarcated by dotted lines, as indicated. Scale bars: 500 mM. (**D**) High-power images of the OSOM. Scale bars: 100 mM.

To explore the cellular localization of RAR signaling after Rhabdo-AKI, we costained the RARE-LacZ reporter kidneys for LacZ and tubular segment and inflammatory cell markers. The majority of LacZ^+^ cells were localized in LTL^+^ PTECs in the cortex and OSOM (Figure 3, A and B). Less than 1% of LacZ^+^ cells were Kim1^+^ PTECs 24 hours after injury, increasing to 17% in the OSOM and 21% in the cortex 72 hours after injury (Figure 3, C and D). This contrasts with our data in mice after IRI-AKI (41), where greater than 40% of LacZ^+^ cells were localized in Kim1^+^ tubules in the OSOM 24 hours after injury (Supplemental Figure 4). Compared with PTECs, there was a lower proportion of LacZ^+^ cells in THP1^+^ TAL cells (Supplemental Figure 5, A and B) and in F4/80^+^ renal macrophages in the OSOM after Rhabdo-AKI (Supplemental Figure 5, C and D). In addition, there were only a small number of LacZ^+^ cells in AQP2^+^ CD cells 1–7 days after Rhabdo-AKI (Supplemental Figure 5, E and F). The percentage of LacZ^+^ AQP2^+^ CD cells increased in the OSOM by day 14 (Supplemental Figure 5, E and F). However, at this time point, the total numbers of LacZ^+^ cells in the kidney returned to near baseline levels (Figure 2, D and E) so that the total number of AQP2^+^ CD cells with activation of RAR signaling 14 days after injury was low. These findings are consistent with human data showing dominant activation of RAR signaling in PTECs from patients with SA-AKI, but they also indicate that there are differences in the kinetics and localization of RAR signaling in different AKI models.

### Inhibition of RAR signaling in PTECs protects against ATI.

**Figure 4:**
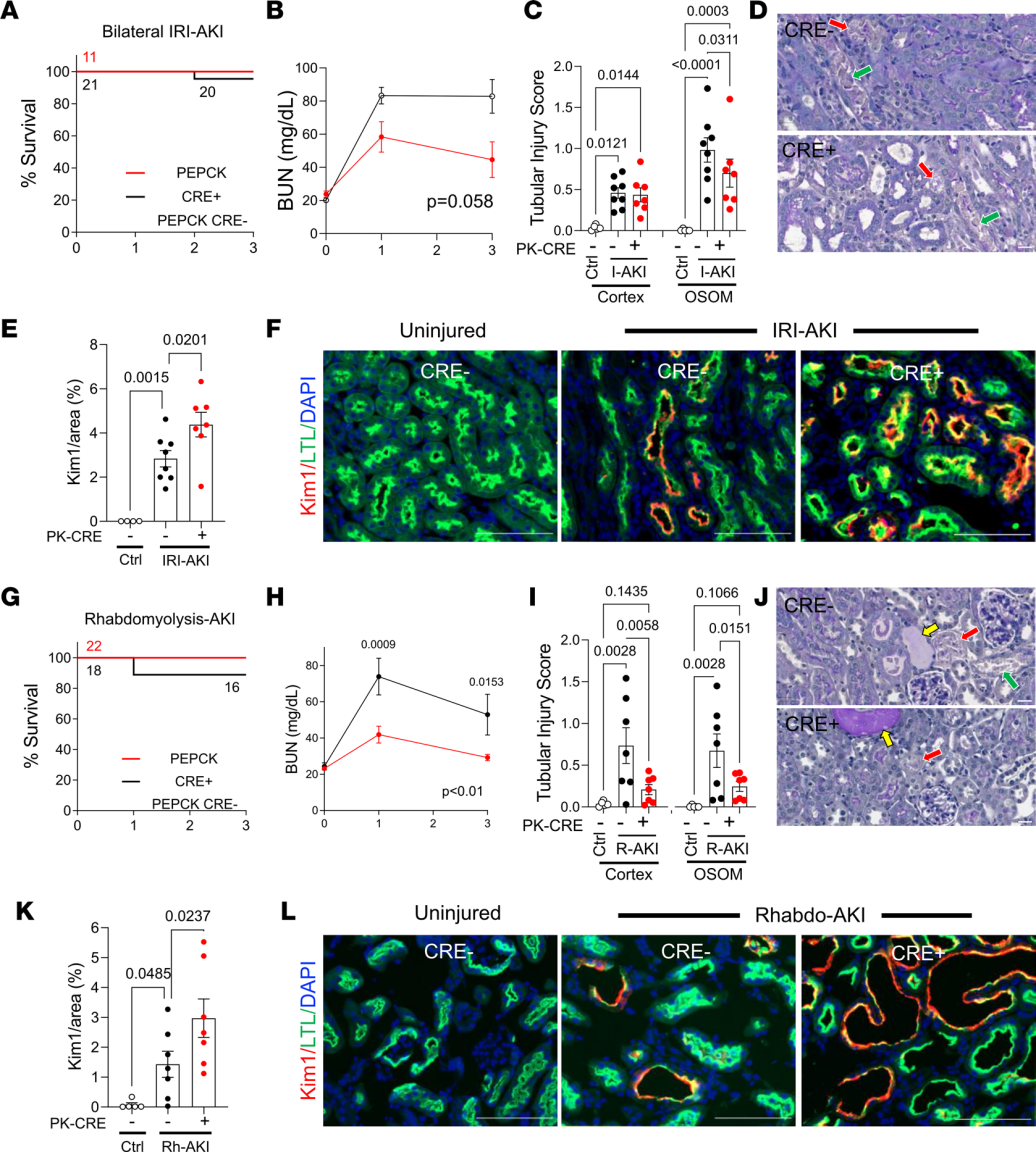
Inhibition of RAR signaling in PTECs protects against AKI but also increases Kim1 expression in PTECs. *PEPCK Cre^+^DN-RAR* (PTEC DN RAR) mice and *Cre^–^* controls mice underwent bilateral IRI-AKI, or Rhabdo-AKI, and kidneys were harvested after 3 days. (**A**–**F**) Bilateral IRI-AKI. (**A**) Survival curves. Mouse numbers indicated after each event. (**B**) BUN time course. Mouse numbers are indicated in **A**. (**C**) Tubular injury scores in the cortex and OSOM. (**D**) PAS-stained kidney images from the OSOM. Red arrows indicate necrosis; green arrows indicate detached epithelial cells. (**E**) Quantification of Kim1 staining. (**F**) Kim1 and LTL staining in OSOM. (**G**–**L**) Rhabdo-AKI. (**G**) Survival curves. (**H**) BUN time course. (**I**) Tubular injury scores. (**J**) PAS-stained kidney images. Red and green arrows, as described above; yellow arrows indicate tubular casts. (**K**) Quantification of Kim1 staining in the OSOM. (**L**) Kim1 and LTL staining in OSOM. Scale bars: 20 mM (**D** and **J**), 100 mM (**F** and **K**). **B** and **H** used 2-way ANOVA; *P* values are indicated, and if *P* < 0.05, *q* values are shown for between-group comparisons corrected for repeated testing at the indicated time points. **C**, **E**, **I**, and **K** used 1-way ANOVA, and if *P* < 0.05, *q* values are shown for between-group comparisons corrected for repeat testing.

To explore the functional role of PTEC RAR signaling after AKI, we crossed *Rosa26-LSL-RARaT403X* (*R26R-DN RAR*) mice (33) — which express a Cre-activated, truncated dominant-negative RARa mutation (which inhibits RARa, -b, and -g) — with *PEPCK-Cre* mice (53), which induce efficient Cre-dependent recombination in PTECs (41). The *R26R-DN RAR* allele was bred to homozygosity to inhibit PTEC RAR signaling (PTEC DN RAR mice), as described (33). In previous studies, we showed that PTEC DN RAR mice have normal kidneys but have increased renal Kim1 expression after IRI-AKI compared with *Cre^–^* controls (41). This was thought to reflect increased PTEC injury but notably was not associated with increased tubular injury scores in PAS-stained sections (41). We now show that there was a nonsignificant improvement in renal function and an increase in *Kim1* mRNA and Kim1 staining extending from the OSOM to the cortex of PTEC DN RAR kidneys 3 days after IRI-AKI (Figure 4, A, B, E, and F, and Supplemental Figure 6, A and C). This was unexpectedly associated with reduced tubular injury, as determined by tubular injury scores (Figure 4, C and D), and a reduction in *NGal/Lcn2 mRNA* expression, which is expressed by injured TAL, CD, and distal tubules but not PTECs (Supplemental Figure 6A) (54). To explore this further, we evaluated the severity of Rhabdo-AKI in PTEC DN RAR mice. Here we saw a significant improvement in renal function 3 days after injury (Figure 4, G and H). This was associated with reduced tubular injury in the cortex and in the OSOM of PTEC DN RAR mice (Figure 4, I and J) and with increased *Kim1* expression and Kim1 staining. There was, however, no significant change in renal NGal mRNA expression compared with Cre^–^ controls (Supplemental Figure 6, B and D, and Figure 4, K and L).

To determine whether inhibition of RAR signaling in PTECs affects tissue repair after AKI, we evaluated long-term renal outcomes in PTEC DN RAR mice after IRI and Rhabdo-AKI. We used a model of unilateral IRI-AKI with delayed contralateral nephrectomy (IRI-DN), compared with nephrectomy only controls (Nx). This model allows for the induction of severe AKI and results in long-term fibrosis and reduced renal function (55). With this model, there was a reduction in BUN at early time points after Nx in PTEC DN RAR mice but no difference in transdermal glomerular filtration rates (tGFR) or renal fibrosis 28 days after injury compared with *Cre^–^* controls (Supplemental Figure 7, A–C). Likewise, there was improved survival and reduced BUN in the first 3 days after Rhabdo-AKI but no differences in BUN or tubular injury scores 14 days after injury in PTEC DN RAR mice (Supplemental Figure 7, D–F). These studies indicate that the principal effect of inhibiting RAR signaling in PTECs is to reduce early tubular injury and not to reduce fibrosis or improve long-term renal function.

### Inhibition of RAR signaling promotes dedifferentiation and metabolic reprogramming of PTECs.

**Figure 5:**
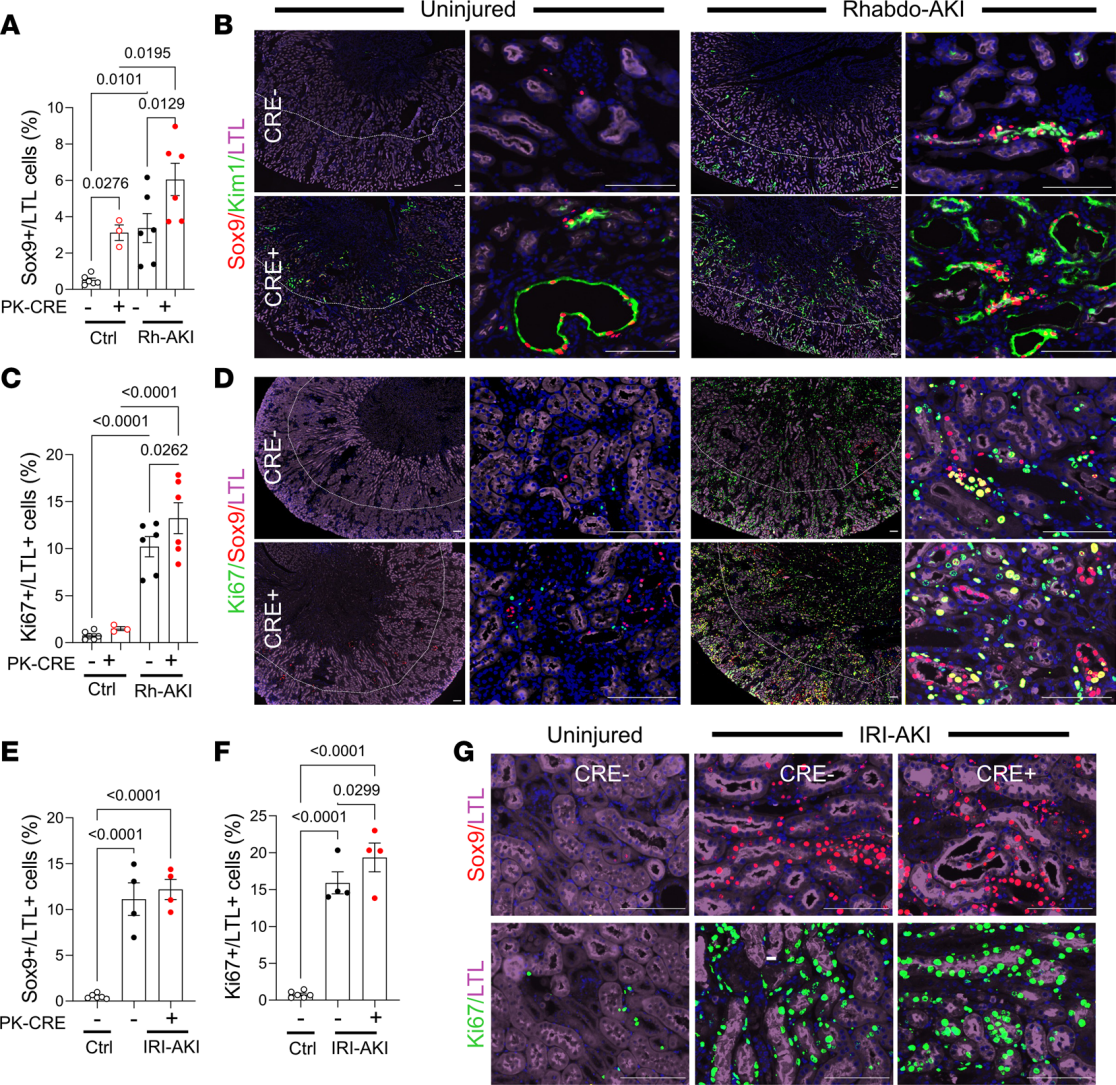
PTEC DN RAR mice have increased expression of PTEC dedifferentiation and proliferation markers after AKI. PTEC DN RAR mice underwent Rhabdo-AKI or bilateral IRI-AKI, and kidneys were harvested after 3 days. (**A**–**D**) Rhabdo-AKI. (**A**) The percentage of LTL^+^ PTECs that are Sox9^+^ in the OSOM. (**B**) Sox9, Kim1, and LTL staining. Left panels are low magnification, showing OSOM staining extending into the cortex. Right panels show higher magnification of the OSOM. (**C**) The percentage of LTL^+^ PTECs that are Ki67^+^ in the OSOM. (**D**) Ki67, Sox9, and LTL staining. (**E**–**G**) Bilateral IRI-AKI. (**E** and **F**) The percentage of LTL^+^ cells that are Sox9^+^ and Ki67^+^ in the OSOM 3 days after IRI-AKI. (**G**) Sox9 and Ki67 staining in the OSOM. Scale bars: 100 mM. (**A**, **C**, **E**, **F**) 1-way ANOVA; if *P* < 0.05, then *q* values are shown for between group comparisons corrected for repeated testing.

Because Kim1 expression usually correlates with the severity of tubular injury after AKI (20–23), our finding that increased Kim1 expression was associated with reduced tubular injury in PTEC DN RAR mice was unexpected and suggests that inhibition of RAR signaling results in the upregulation of Kim1 expression in less-injured PTECs after AKI. This is supported by the observation that there was an extension of Kim1 staining into the cortex after IRI-AKI in PTEC DN RAR mice (Supplemental Figure 6C), a region of the kidney that is relatively spared from injury compared with the OSOM in this model (14). Since Kim1 is expressed by dedifferentiated and proliferating PTECs after AKI (19, 20), we sought to determine whether increased Kim1 expression in PTEC DN RAR mice was associated with activation of a more generalized dedifferentiation program in PTECs. As anticipated, there was a marked reduction in the numbers of cells staining with Lotus tetragonolobus lectin (LTL), an apical marker of differentiated PTECs, 3 days after Rhabdo- and IRI-AKI compared with uninjured controls (Supplemental Figure 8 and Figure 5, B and D). However, there were also reduced numbers of LTL staining cells in the OSOM of PTEC DN RAR kidneys 3 days after Rhabdo-AKI compared with *Cre^–^* injured controls (Supplemental Figure 8 and Figure 5, B and D). In addition, we found increased numbers of Sox9^+^ PTECs (defined by LTL and DAPI staining nuclei), a transcription factor that is expressed by dedifferentiated and regenerative PTECs after AKI (56, 57), and Ki67^+^ PTECs, a marker of active cell proliferation, in PTEC DN RAR mice after Rhabdo-AKI (Figure 5, A–D). In the IRI-AKI model, we found a similar increase in Ki67^+^ PTECs but without an increase in Sox9 staining, and we found a nonsignificant decrease in LTL staining in PTEC DN RAR mice compared with Cre^–^ controls (Supplemental Figure 8 and Figure 5, E–G). In Rhabdo-AKI, the majority of Sox9^+^ PTECs were also Kim1^+^ both in *PEPCK Cre^+^* and *Cre^–^* mouse kidneys (Figure 5B).

**Figure 6:**
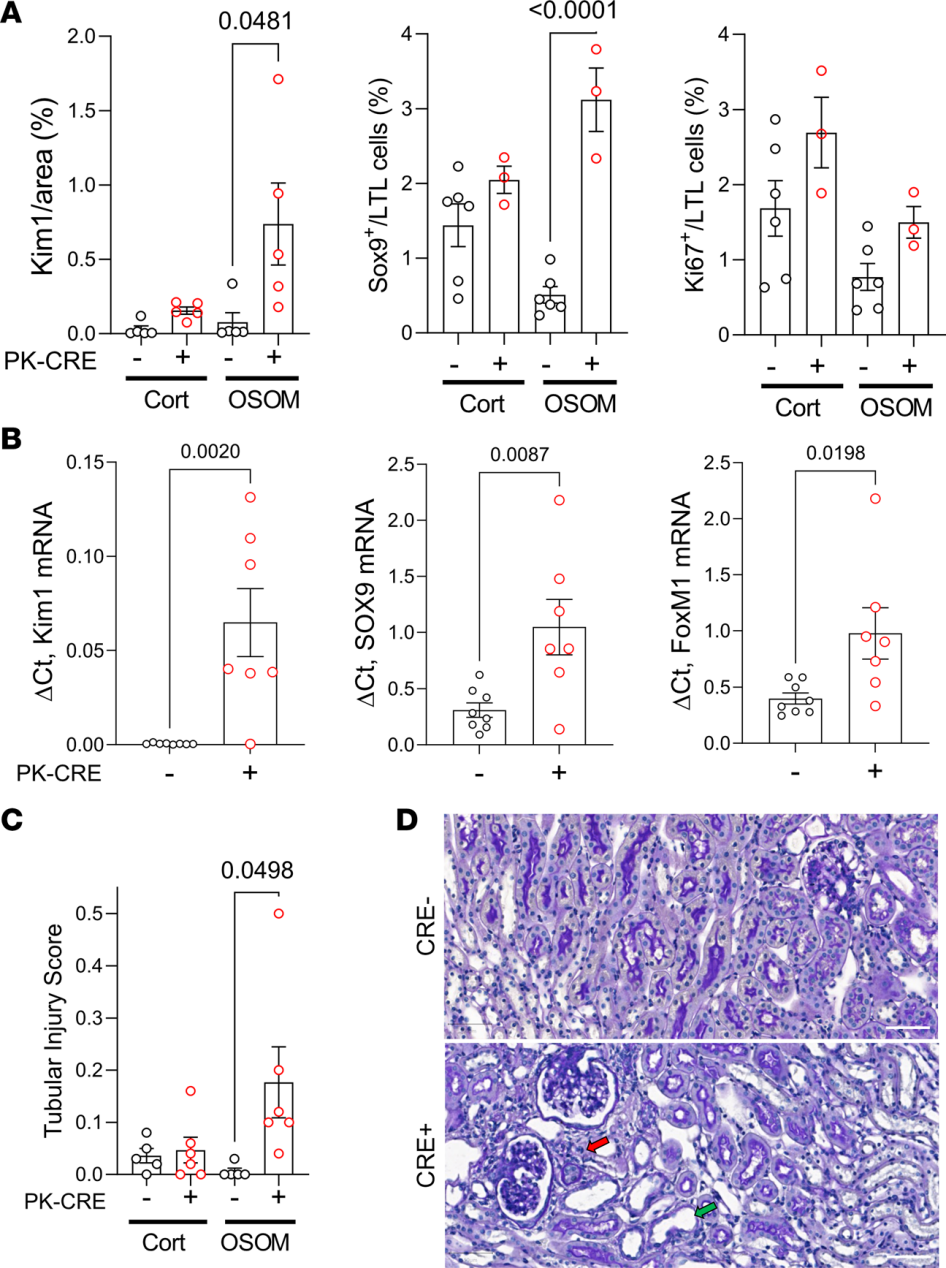
Increased expression of Kim1 and dedifferentiation markers in uninjured PTEC DN RAR mouse kidneys. (**A**) The percentage of the area of Kim1 staining, and the percentage of LTL^+^ PTECs that are Sox9^+^ or Ki67^+^ in the cortex and OSOM of uninjured *PEPCK Cre^+^* and *Cre^–^* mice. Ki67, Sox9, and Kim1 staining in uninjured *Cre^+^* and *Cre^–^* kidneys shown in Figure 5, B and D. (**B**) Renal *Kim1*, *Sox9*, and *FoxM1* mRNA expression. (**C**) Tubular injury scores. (**D**) PAS-stained images showing areas with flattened epithelia (green arrow) and peritubular inflammatory cells (red arrow) in the OSOM of uninjured *Cre^+^* and *Cre^–^* kidneys. Scale bars: 50 mM. The OSOM Sox9 and Ki67 data in **A** are the same as the uninjured controls in Figure 5, A and C. **A**–**C** used *t* tests, with *P* values comparing *PEPCK Cre^+^* and *Cre^–^* mice.

To examine this in more detail, we evaluated the same markers in uninjured PTEC DN RAR kidneys. In the absence of injury, the area staining Kim1^+^ was lower than after AKI (Figure 4, E and K). However, there was increased Kim1 and decreased LTL staining in the OSOM of uninjured PTEC DN RAR mice compared with Cre^–^ controls (Figure 6A, Figure 5B, and Supplemental Figure 8). Vimentin, another marker of dedifferentiated PTECs (19), was restricted to a subset of Kim1^+^ as well as a smaller number of Kim1^–^PTECs in uninjured PTEC DN RAR kidneys (Supplemental Figure 9, A and B). There were also increased numbers of Sox9^+^, but not Ki67^+^ PTECs (Figure 5, A–D, and Figure 6A), and increased expression of *Kim1*, *Sox9*, and *FoxM1* mRNAs — the latter of which is also upregulated in dedifferentiated, proliferating PTECs after IRI-AKI (31) — in uninjured PTEC DN RAR mouse kidneys compared with Cre^–^ controls (Figure 6B). Scoring for histological features of ATI suggests there was also increased tubular injury in uninjured PTEC DN RAR mice (Figure 6, C and D). However, unlike AKI, where tubular injury is characterized by the presence of tubular necrosis, detached epithelium, and tubular casts (Figure 4, D and J), in uninjured PTEC DN RAR mice, the tubular injury score was driven by the detection of dedifferentiated, flattened epithelial cells, and the presence of interstitial inflammatory cells (Figure 6D). These data indicate that inhibition of RAR signaling results in PTEC dedifferentiation in PTEC DN RAR mouse kidneys.

**Figure 7:**
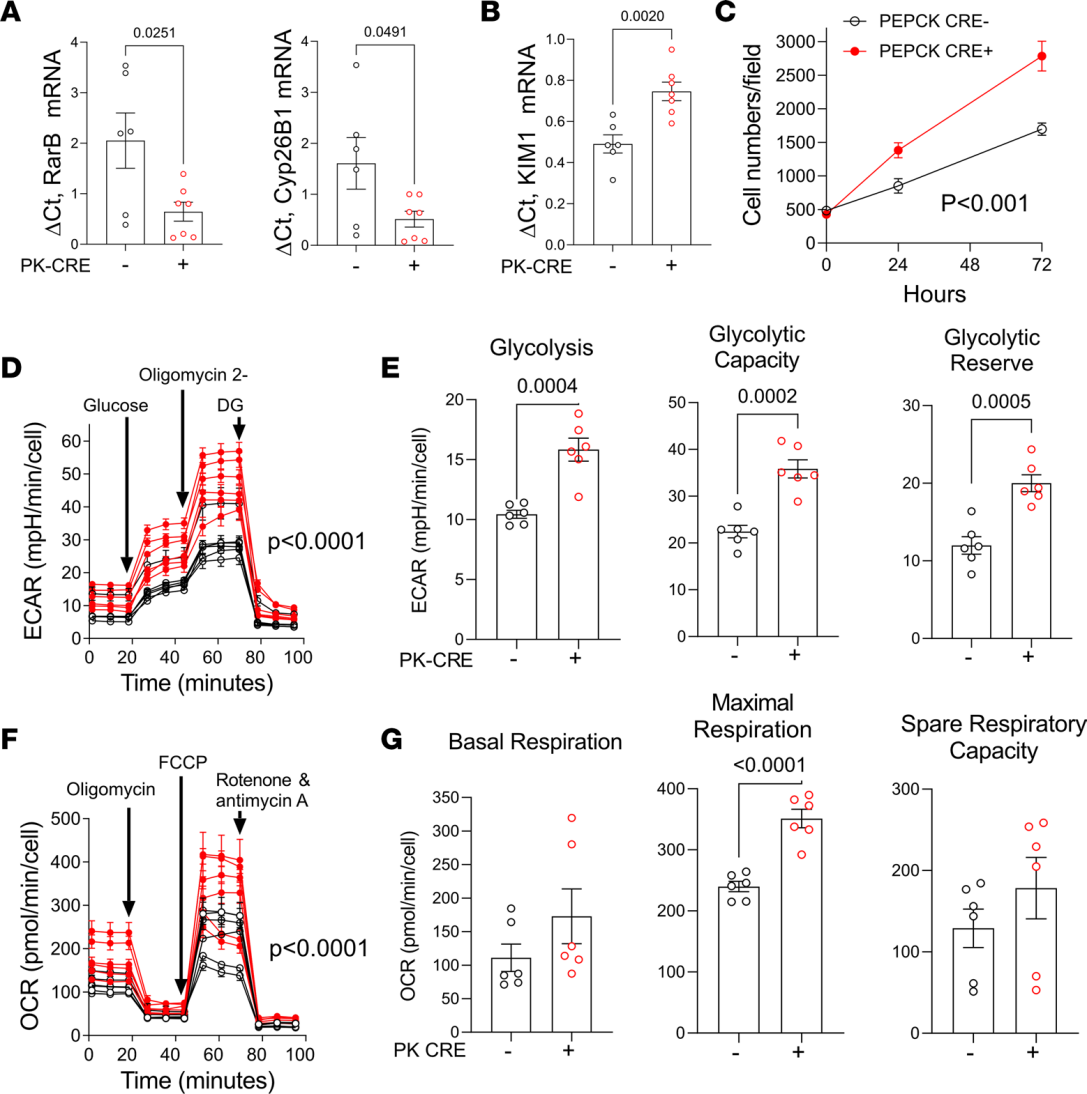
Inhibition of RAR signaling increases proliferation, glycolysis, and oxidative phosphorylation in cultured PTECs. Data are shown for replicates performed on separate preparations of primary PTECs isolated from different mice. (**A**) RAR target genes. (**B**) Expression of *Kim1* mRNA. (**C**) Cell growth. Cell numbers/10***×*** microscopy field at the indicated time points in PTECs from 4 *PEPCK Cre^+^* and 4 *Cre^–^* mice. (**D**) Glycolytic stress tests on PTECs from 6 *Cre^+^* (red) and 6 *Cre^–^* (black) mice. (**E**) Glycolytic rate, maximal glycolytic capacity, and glycolytic reserve. (**F**) Mitochondrial stress test. (**G**) Basal mitochondrial respiration, maximal mitochondrial respiration, and spare respiratory capacity. **A**, **E**, and **G** used *t* tests, with *P* values comparing *PEPCK Cre^+^* and *Cre^–^* PTECs. **D** and **F** used 2-way ANOVA, with *P* values comparing *PEPCK Cre^+^* and *Cre^–^* PTECs over time.

**Figure 8:**
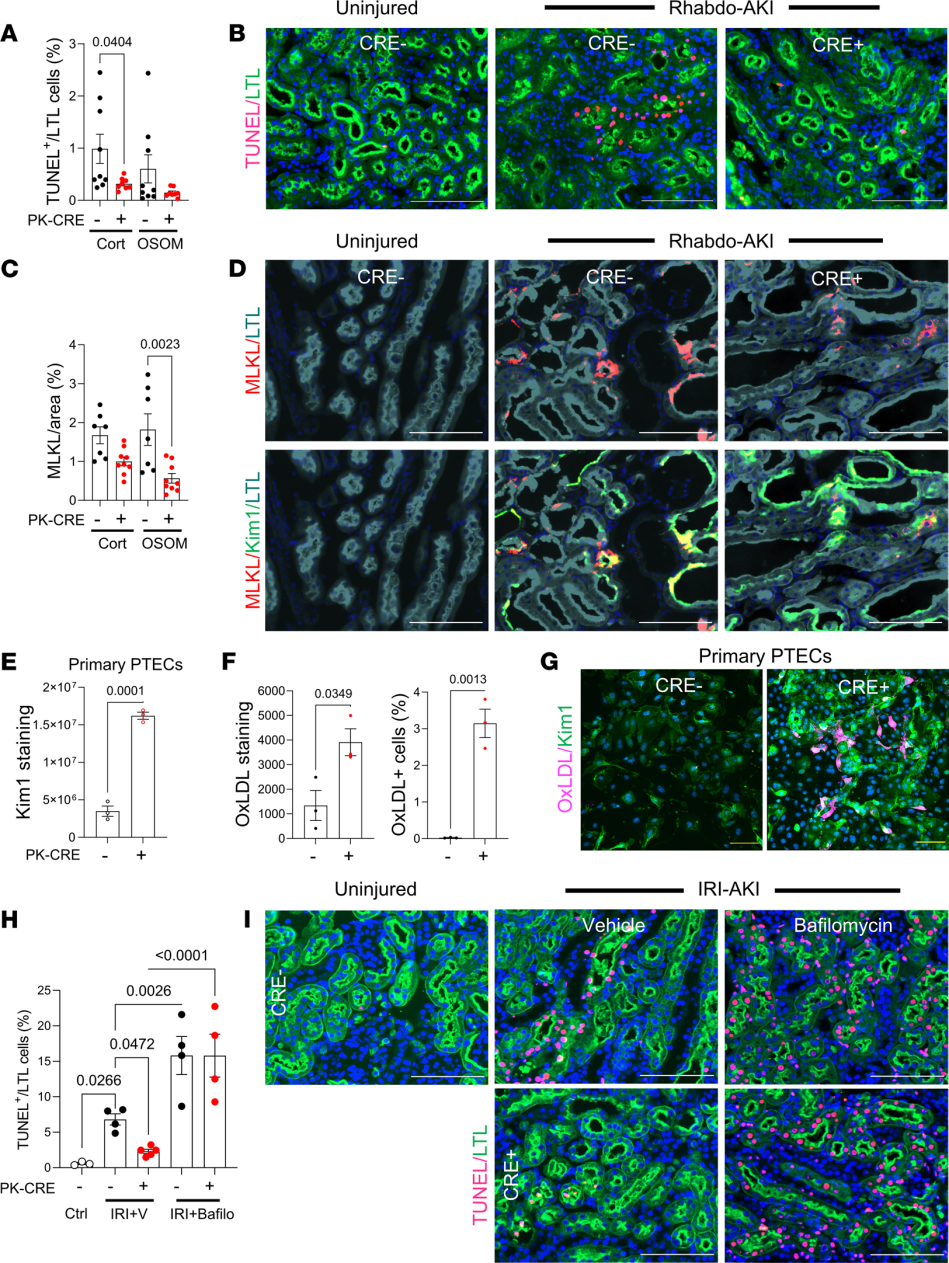
Increased efferocytosis in PTEC DN RAR mice after AKI. (**A**–**E**) Apoptosis in PTEC DN RAR mice 3 days after Rhabdo-AKI. (**A**) The percentage of TUNEL^+^LTL^+^ cells in the cortex and OSOM. (**B**) TUNEL and LTL staining in the OSOM. (**C**) Percentage of MLKL staining. (**D**) MLKL with LTL and Kim1 staining in the OSOM. (**E**–**G**) Kim1 expression and endocytic activity in cultured PTECs. (**E**) Kim1 fluorescence intensity. (**F**) PTECs uptake of fluorescently conjugated oxidized LDL (Ox-LDL). Fluorescence intensity and numbers of PTECs taking up Ox-LDL. (**G**) Kim1 staining and Ox-LDL uptake. (**H** and **I**) Lysosomal clearance of apoptotic cells in PTEC DN RAR mice. (**H**) PTEC DN RAR mice after bilateral IRI-AKI pretreated with bafilomycin A1. Kidneys harvested at 24 hours. The percentage of TUNEL^+^LTL^+^ cells in the OSOM. (**I**) TUNEL and LTL staining. Scale bars: 100 mM (**B**, **D**, **E**, and **J**), 20 mM (**H**). **A**, **C**, **E**, and **F** used *t* tests, with *P* values comparing PEPCK *Cre*^+^ and *Cre*^–^ mice. **H** used 1-way ANOVA, with *P* < 0.05 considered significant; *q* values are shown for between-group comparisons corrected for repeated testing.

Next, we evaluated the functional characteristics of primary PTECs isolated from PTEC DN RAR and *Cre^–^* control mouse kidneys to determine whether they demonstrate characteristics of dedifferentiated, proliferating epithelium. Early passage (P2/3) cells used for our studies were enriched for PTECs with > 80% expression of PTEC markers Kim1 and AQP1 and less than 1% αSma^+^ myofibroblast contamination (Supplemental Figure 10A). As anticipated, we found decreased expression of the RAR target genes, *Rarb* and *Cyp26b1* (Figure 7A), and increased expression of *Kim1* mRNA (and protein) in primary PTECs derived from PTEC DN RAR mice compared with Cre^–^ controls (Figure 7B and Figure 8, E and G). Cell counting also revealed that PTECs from PTEC DN RAR mice have more rapid growth rates in culture than *Cre^–^* controls (Figure 7C). This is consistent with our data showing increased numbers of Ki67^+^ PTECs in PTEC DN RAR mice after AKI.

AKI is associated with mitochondrial damage giving rise to reduced mitochondrial oxidative metabolism and with increased glycolysis in dedifferentiated, proliferating PTECs (58–60). Moreover, compared with other tubular segments, PTECs are thought to be particularly susceptible to injury because they have relatively low levels of glycolysis and are therefore dependent on mitochondria to generate ATP for solute transport functions and survival (58, 61). To explore the role of RAR signaling in these processes, we used Seahorse assays to compare cellular metabolism of primary PTECs isolated from PTEC DN RAR and *Cre^–^* control mice. PTECs from PTEC DN RAR mice have increased resting glycolysis, maximal glycolytic capacity, and glycolytic reserve (Figure 7, D and E) but also have increased maximal respiratory capacity. There were no differences in basal respiration or spare respiratory capacity from mitochondrial oxidative metabolism compared with *Cre^–^* control PTECs (Figure 7, F and G). This is not associated with an increase in *Pgc1a* (which improves mitochondrial function; ref. 62) or *Hk2* (required for glucose metabolism) mRNAs, but it was associated with increased expression of *PfkB*, a rate-limiting step for glycolysis (Supplemental Figure 10B) (59). These findings suggest that PTECs from PTEC DN RAR mice have metabolic features of dedifferentiated and regenerating PTECs (increased glycolysis), without evidence of injury-associated mitochondrial damage, since they have increased maximal respiratory capacity from mitochondrial oxidative metabolism. These findings also suggest that inhibition of RAR signaling induces metabolic reprogramming that could enhance the regenerative capacity of PTECs while protecting them from the damaging effects of ATP depletion in AKI.

### Kim1-dependent efferocytosis and suppression of inflammatory cell activation after AKI in PTEC DN RAR mice.

Increased Kim1 expression in PTEC DN RAR mice may reduce tubular injury by enhancing Kim1-dependent apoptotic cell clearance, or efferocytosis (24–28). Consistent with this, there was a reduction in TUNEL^+^ PTECs (Figure 8, A and B) and a reduction in the expression of mixed-lineage kinase domain–like protein (MLKL; a key necroptosis effector protein) (63) in PTEC DN RAR kidneys compared with *Cre^–^* controls after Rhabdo-AKI (Figure 8, C and D). MLKL is membrane associated and largely colocalizes with apical Kim1 in *Cre^–^* control but not in PTEC DN RAR kidneys (Figure 8D and Supplemental Figure 11). Since membrane-associated, apical MLKL is a terminal effector of necroptotic cell death in PTECs (64), these findings suggest that reduced PTEC apoptosis is associated with reduced cellular necrosis. These findings would occur if there was increased Kim1-dependent apoptotic cell clearance or reduced apoptotic cell death. To distinguish between these possibilities, we first sought to determine whether there was increased Kim1 scavenging function in PTEC DN RAR mice. In addition to its role as a phosphatidyl-serine receptor, Kim1 mediates the endocytic uptake of oxidized lipoproteins that are also exposed on the surface of apoptotic cells (24, 27). Using primary PTECs isolated from PTEC DN RAR mice, which also show increased Kim1 staining in culture (Figure 8, E and G), we found that there is increased cellular uptake of fluorescently conjugated oxidized LDL (Figure 8, F and G). This confirms that PTECs from PTEC DN RAR mice have increased Kim1 functionality. We next evaluated whether blocking lysosomal degradation of apoptotic cells, which is required for Kim1-dependent clearance of apoptotic cells (27), affects PTEC apoptosis in PTEC DN RAR mice after IRI-AKI. Like Rhabdo-AKI, there is a reduction in TUNEL^+^ PTECs 24 hours after bilateral IRI-AKI in PTEC DN RAR compared with *Cre^–^* control injured kidneys (Figure 8, H and I). To block lysosomal clearance of apoptotic cells, we treated mice with the vacuolar H^+^ ATPase inhibitor, bafilomycin A1, as described (27). By preventing lysosomal clearance of apoptotic cells that have been phagocytosed by PTECs via Kim1-dependent efferocytosis, treatment with bafilomycin increases the number of apoptotic nuclei detected in PTECs after AKI (Figure 8, H and I). However, bafilomycin also reverses the effect of PTEC DN RAR mice on apoptotic PTECs 24 hours after IRI-AKI (Figure 8, H and I). Since apoptotic cells are normally degraded and undetectable by TUNEL assay within 6 hours of being phagocytosed by PTECs (27), these findings indicate that reduced detection of apoptotic PTECs after AKI results from increased efferocytosis, rather than reduced PTEC apoptotic cell death, in PTEC DN RAR mice.

**Figure 9:**
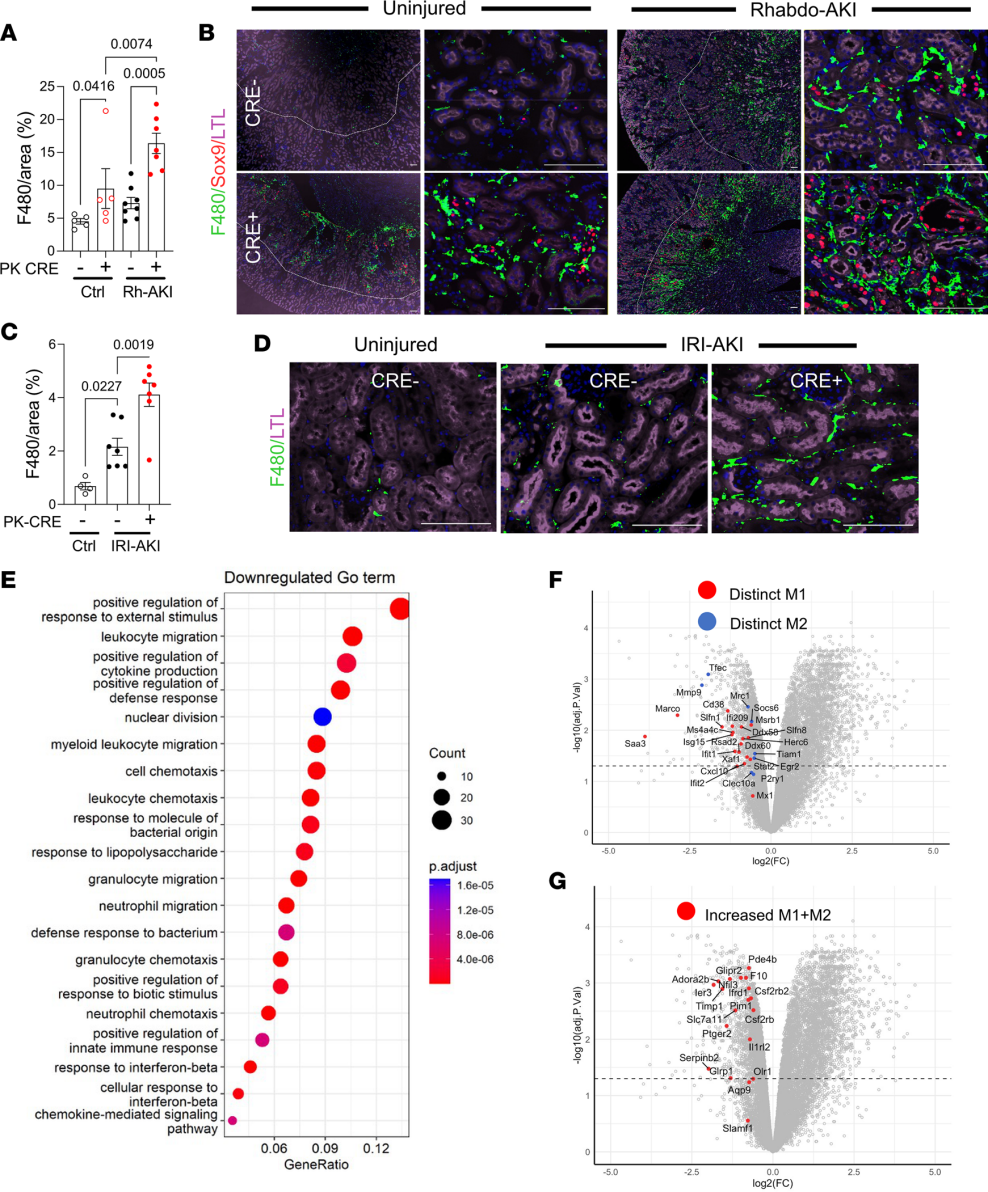
Increased renal macrophages with a reduced proinflammatory signature in PTEC DN RAR mice after AKI. PTEC DN RAR mice underwent Rhabdo- or bilateral IRI-AKI, and kidneys were harvested after 3 days. (**A** and **B**) Rhabdo-AKI. The percentage of F4/80 area staining in the OSOM, and images showing F4/80, Sox9, and LTL staining. Left panels show F4/80 staining largely restricted to the OSOM. Right panels show higher magnification of the OSOM. (**C** and **D**) Bilateral IRI-AKI. Quantification, and F4/80 and LTL staining in the OSOM. (**E** and **F**) Expression of inflammatory markers in CD11B^+^ cells after IRI-AKI. PTEC DN RAR mice underwent bilateral IRI-AKI, and bulk RNA-Seq was performed on renal CD11B^+^ cells 3 days after injury. (**E**) GSEA of downregulated genes using GO data sets. (**F** and **G**) GSEA for proinflammatory (“M1”) and antiinflammatory (“M2”) gene sets in the RNA-Seq data. Set size=no. of genes from each gene set that are represented. NES=normalized expression score for the gene set sizes. Volcano plots showing fold change in expression of core enrichment gene from the CD11B^+^ RNA-Seq data set. Dotted line indicates *P* < 0.05. **A** and **C** used 1-way ANOVA, with *P* < 0.05 considered significant; *q* values shown for between-group comparisons corrected for repeated testing. Scale bars: 100 μM.

Apoptotic cell clearance by Kim1-mediated efferocytosis suppresses the activation of macrophage-dependent inflammatory responses after AKI (27). We therefore asked whether there was a reduction in renal macrophage activation in PTEC DN RAR mice after AKI. Unexpectedly, we saw an increase in staining with F4/80 antibodies, a marker of tissue macrophages (65), in PTEC DN RAR mice after both Rhabdo-AKI and IRI-AKI (Figure 9, A–D). F4/80 staining was also increased in uninjured PTEC DN RAR mouse kidneys (Figure 9, A and B). F4/80 staining was closely associated with Sox9^+^ PTECs in both uninjured PTEC DN RAR mice and injured mice after Rhabdo-AKI (Figure 9B). To explore this further, we performed RNA-Seq on CD11B^+^ mononuclear cells isolated from PTEC DN RAR and *Cre^–^* control mouse kidneys 3 days after IRI-AKI. Principal component analysis (PCA) of these data sets shows clear separation of the 2 genotypes along the first dimension, accounting for 57% of the variation between samples (Supplemental Figure 12). GSEA using the Gene Ontology (GO) data sets demonstrated a downregulation of proinflammatory pathways in PTEC DN RAR versus *Cre^–^* control CD11B^+^ cells in the kidney after IRI-AKI (Figure 9E and Supplemental Table 3). To evaluate this further, we performed GSEA using published data sets for validated proinflammatory (M1 activated) and antiinflammatory (M2 activated) macrophage genes generated by transcriptional profiling of unstimulated mouse bone marrow–derived macrophages (BMDMs) versus BMDMs stimulated with LPS and IFN-γ (to induce classical M1 activation) versus IL-4 (to induce antiinflammatory, M2 macrophages) (Supplemental Table 4; ref. 66). The same data sets have been used to characterize renal macrophage populations after AKI (67). There is a significant downregulation of genes that are uniquely upregulated in M1 activated macrophages (“distinct M1 up”) but also of genes that are increased in both M1 and M2 macrophages compared with untreated control BMDMs (“increased M1+M2”) and of genes increased in M1 activated macrophages compared with untreated control BMDMs (“M1 up”) (Table 2; Figure 9, F and G; and Supplemental Table 4). There was, however, no difference in expression of genes upregulated in M2 macrophages (“distinct M2 up” and “M2 up”) (Table 2). These findings indicate that there was primarily a suppression of proinflammatory macrophage activation markers in the kidneys of PTEC DN RAR mice after IRI-AKI.

**Figure 10:**
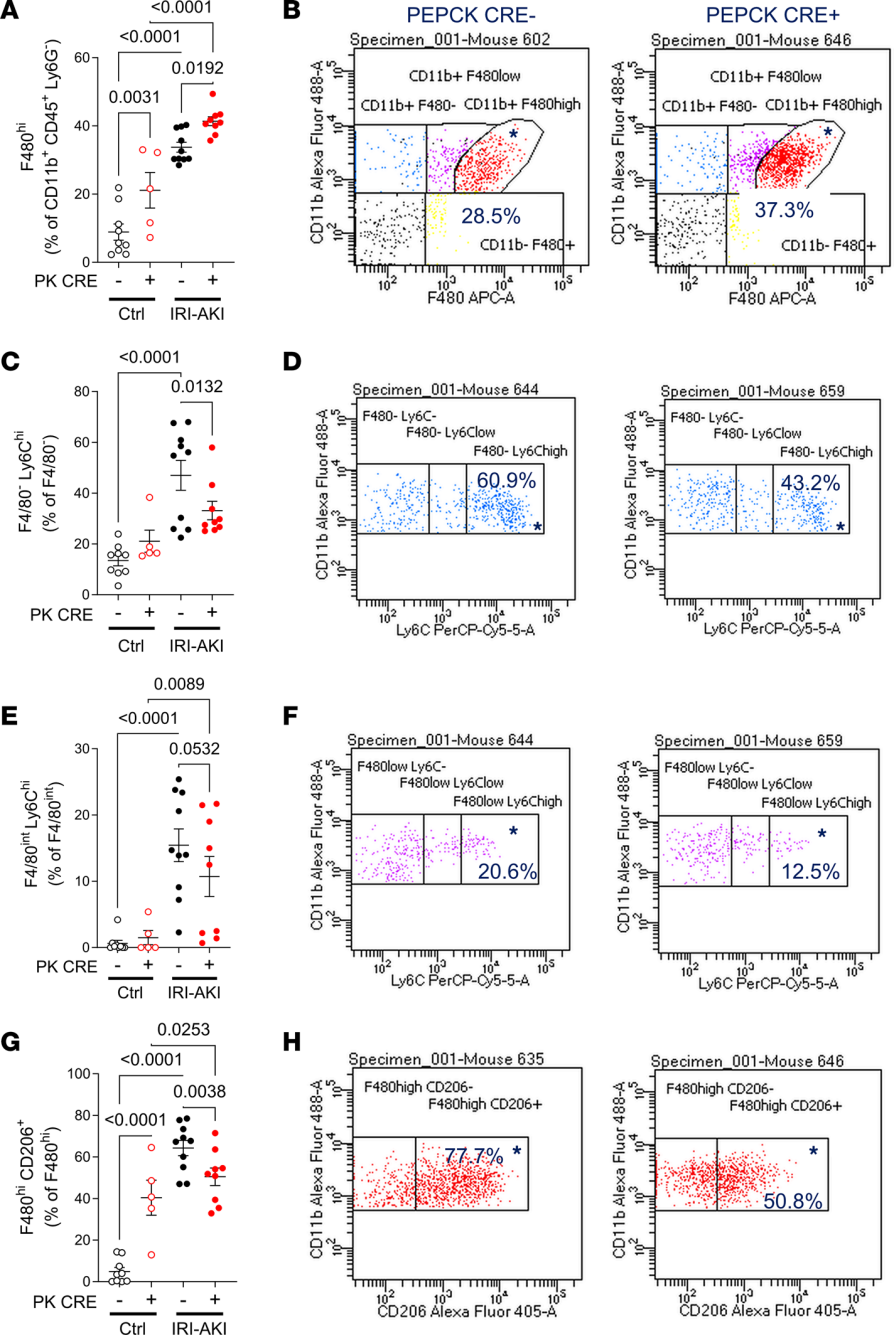
Flow cytometric analysis of renal monocyte/macrophages in PTEC DN RAR mice. Kidneys were harvested from PTEC DN RAR mice 3 days after bilateral IRI-AKI. (**A** and **B**) F4/80^hi^ cells (kidney-resident macrophages). (**A**) F4/80^hi^ cells as the percentage of gated CD11B^+^CD45^+^Ly6G^–^ cells. (**B**) CD11B and F4/80 expression charts in *PEPCK Cre^+^* and *Cre^–^* mice after IRI-AKI (percentage in gated area indicated). (**C**–**F**) Ly6C^hi^ cells (inflammatory monocyte/macrophages). (**C** and **E**) F4/80^–^ (infiltrating monocytes) Ly6C^hi^ cells and F4/80^int^ (BM-derived macrophages) Ly6C^hi^ cells, as the percentage of gated F4/80^–^ and F4/80^int^ cells, respectively. (**D** and **F**) CD11B and Ly6C expression charts. (**G** and **H**) CD206/mannose receptor expression (an M2 activated macrophage marker). (**G**) CD206^+^ cells as the percentage of gated F4/80^hi^ cells. (**H**) CD11B and CD206 expression charts. **A**, **C**, **E**, and **G** used 1-way ANOVA, with *P* < 0.05 considered significant; *q* values shown for between-group comparisons corrected for repeated testing.

While CD11B^+^ cells are enriched for renal monocyte/macrophages, they include a number of other cell types, including NK cells, granulocytes, and B cells (68). To further define the phenotype of renal monocyte/macrophages in PTEC DN RAR mice, we performed flow cytometric analysis using a panel of mononuclear cell markers (Supplemental Table 5E). CD45^+^ renal leukocytes were selected, while granulocytes (using Ly6G staining) and CD11B^–^ cells were excluded. Gating was then set to distinguish between F4/80^–^ (early infiltrating monocytes), F4/80^int^ (BM-derived renal macrophages), and F4/80^hi^ (kidney-resident macrophages [KRM]) cells, as described (Supplemental Figure 13) (67, 69). There was an expected increase in total CD45^+^, CD11B^+^, and CD45^+^CD11B^+^ nongranulocyte (ly6G^–^) mononuclear cells after IRI-AKI, there was no difference in total cell numbers between *PEPCK Cre^+^* and *Cre^–^* kidneys (Supplemental Figure 14). Consistent with our IF data, we found an increase in F4/80^hi^ cells in uninjured and IRI-AKI kidneys from PTEC DN RAR mice (Figure 10, A and B). KRM cells also express high levels of MHC Class II antigens (MHC-II^hi^) (67). As previously reported (67), we also detected high levels of MHC-II expression in F4/80^hi^ cells from uninjured mice, and there were fewer F4/80^hi^ MHC-II^hi^ cells after injury (Supplemental Figure 15, A and B). However, F4/80^hi^MHC-II^hi^ cells were increased in PTEC DN RAR mice compared with *Cre^–^* controls after AKI. This confirms that the increase in F4/80^hi^ cells in PTEC DN RAR mice is due to an increase in KRMs. To determine whether the expansion of KRMs in PTEC DN RAR mice was due to increased proliferation, we performed pulse labeling with the S-phase marker, EdU. There was a significant increase in EdU^+^F4/80^hi^ cells in PTEC DN RAR compared with *Cre^–^* controls after AKI (Supplemental Figure 15, C and D). This suggests that the increase numbers of F4/80^hi^ KRMs in PTEC DN RAR mouse kidneys arises, at least in part, from an increased rate of proliferation. We also noted a reduction in the cell inflammatory marker, Ly6C, in F4/80^–^ and F4/80^int^ infiltrating monocyte/macrophages and in BMDMs (Figure 10, C–F). Consistent with these findings, there was a reduction in cells staining with an anti-Gr1 antibody (which recognizes activated monocytes/macrophages and granulocytes; ref. 70), but not myeloperoxidase (MPO) (which only recognizes granulocytes; ref. 71) in PTEC DN RAR mice after IRI-AKI (Supplemental Figure 15, G–I). CD206/mannose receptor^+^F4/80^hi^ cells, a marker of M2 activated macrophages (72), were also reduced in PTEC DN RAR kidneys after AKI, and there was a paradoxical increase in CD206^+^F4/80^hi^ KRMs in uninjured PTEC DN RAR kidneys compared with *Cre^–^* controls (Figure 10, G and H). Expansion of M2 macrophages may explain why there is no evidence of tubular injury in uninjured PTEC DN RAR mouse kidneys despite the increase in F4/80^+^ KRMs.

These data indicate that, despite the increase in F4/80^+^ KRMs in PTEC DN RAR mice, there is suppression of proinflammatory monocyte/macrophage activation in the absence of injury and after IRI-AKI. Since Kim1-dependent apoptotic cell clearance results in the suppression of early macrophage-dependent inflammatory responses after AKI (27), this is consistent with our findings of increased Kim1-dependent clearance of apoptotic cells in PTEC DN RAR mice. In addition, since early proinflammatory macrophage responses worsen injury after Rhabdo- and IRI-AKI (73–75), these findings may account for the reduction in severity of AKI after inhibition of RAR signaling in PTECs. This contrasts with an earlier study showing that long-term overexpression of Kim1 in epithelial lineages increases expression of inflammatory markers and increases renal fibrosis (30), and it suggests that while short-term upregulation of Kim1 in PTEC DN RAR mice protects against AKI, long-term Kim1 overexpression results in proinflammatory kidney injury and fibrosis.

## Discussion

Our analysis of human RNA-Seq data (11) shows that there is enrichment for RAR target genes in the kidneys of patients with severe SA-AKI and that these are dominantly expressed by PTECs. The most highly enriched RAR target genes in SA-AKI PTECs are also upregulated in mouse kidneys after Rhabdo-AKI, and there is more widespread activation of RAR signaling in PTECs after Rhabdo- than after IRI-AKI. In this respect, it is notable that circulating cell-free hemoglobin (CFH) in patients with sepsis can induce heme-dependent PTEC injury and exacerbate AKI in mouse models of SA-AKI (76, 77). Since heme injury to PTECs is also the dominant mechanism of injury in Rhabdo-AKI (78, 79), increased reabsorption of CFH and myoglobin from the tubular filtrate in by Megalin-mediated uptake in S1 and S2 PTEC segments that are found throughout the renal cortex (80–83); this may explain the more widespread activation of RAR signaling in SA- and Rhabdo-AKI compared with IRI-AKI, where PTEC injury and RAR activation is more restricted to PTECs in the OSOM (14).

There is also greater improvement in renal function and a more widespread reduction in tubular injury in PTEC DN RAR mice after Rhabdo-AKI than after IRI-AKI. However, PTEC DN RAR mice have reduced tubular injury associated with increased Kim1 expression in both AKI models, suggesting a common mechanism of renal protection. RAR activation extends throughout the cortex and OSOM after Rhabdo-AKI but is more restricted to the OSOM after IRI-AKI (41). This suggests that the effect of inhibiting RAR signaling in PTECs on renal functional outcomes after AKI may be affected by the differences in the extent and cellular distribution of RAR activation between the 2 models. In addition, while RAR signaling is dominantly activated in Kim1^+^ PTECs after IRI-AKI, there is widespread RAR activation in Kim1^–^ PTECs after Rhabdo-AKI. Since Kim1 is a marker of injured PTECs (20–23), this may be because RAR signaling is activated in PTECs with less severe injury after Rhabdo- than IRI-AKI. One consequence of this might be that Kim1-mediated effects in PTEC DN RAR mice are more pronounced after Rhabdo- than IRI-AKI; PTECs with minor injury that would otherwise have been Kim1^–^ are now induced to express Kim1 as a result of loss of RAR signaling. These findings underscore the importance of using more than one model of human disease to understand relevance to cellular pathophysiologies in human AKI (13).

Our finding that PTEC DN RAR mice are protected from AKI was unexpected, since we have previously shown that systemic inhibition of RAR signaling using the pan-RAR inhibitor, BMS493, worsened injury and increased fibrosis after IRI-AKI (41). However, worsening injury was reversed by macrophage depletion, indicating that the detrimental effects of BMS493 are primarily dependent on macrophage-dependent injury and not on the inhibitory effects of BMS493 on PTEC RAR signaling. These findings are consistent with the known antiinflammatory effects of RA on macrophages (44–46, 84) and suggest that RAR signaling in PTECs and macrophages have opposing effects on renal injury after AKI. The protective effect of inhibiting PTEC RAR signaling is associated with increased apoptotic cell clearance and reduced inflammatory renal macrophages. Phagocytic clearance of apoptotic cells, including Kim1-dependent efferocytosis by PTECs after AKI (27), reduces inflammation in organ injuries (85, 86). This may account for the reduction in renal injury in PTEC DN RAR mice after AKI. However, PTECs from PTEC DN RAR mice also express more Sox9 and have increased glycolytic flux associated with increased mitochondrial oxidative. Since increased Sox9 expression (87–89), and increased glycolytic flux and oxidative phosphorylation (59, 60, 62), may have protective effects on injured PTECs, it is possible that inhibition of RAR signaling makes these cells more resilient to injury. In addition, PTECs from DN RAR mice are more proliferative, suggesting that surviving PTECs may have more robust regenerative responses in PTEC DN RAR mice. If the dominant mechanisms by which PTEC DN RAR mice are protected from AKI are mediated by cytoprotection and/or increased PTEC repair, we would have anticipated that (a) reduced apoptosis in PTEC DN RAR mice would persist after inhibition of lysosomal degradation of apoptotic cells with bafilomycin and/or (b) long-term renal outcomes would improve as a result of increased repair. However, bafilomycin increased apoptotic PTECs in PTEC DN RAR mice with IRI-AKI, and there was no protective effect on long-term renal outcomes in PTEC DN RAR mice after IRI- and Rhabdo-AKI. Therefore, while definitive genetic evidence using Kim1 mutant mice would be required to prove this hypothesis, our findings suggest that, while additional ameliorating mechanisms maybe involved, the dominant mechanism by which inhibition of PTEC RAR signaling protects against AKI is via the increase of Kim1-dependent efferocytosis.

In general, renal macrophages increase with worsening injury, and macrophage-depletion studies indicate that early increases in renal macrophages worsen injury after AKI (73–75). Moreover, genetic studies have shown that loss of Kim1 function or expression increases renal macrophage numbers after AKI (27, 28). On this basis, we had anticipated that reduced renal injury in PTEC DN RAR mice would have been associated with reduced, not increased, renal macrophage numbers. However, prior studies using a conditional genetic approach to overexpress Kim1 in renal epithelium show that persistent Kim1 expression increases inflammatory renal macrophages and causes progressive renal fibrosis (30). This has been used to support the hypothesis that persistent Kim1 expression in surviving PTECs that have failed to undergo productive repair promotes renal inflammation and fibrosis (30). However, this is also consistent with our finding that upregulation of Kim1 in PTEC DN RAR mice is associated with increased renal macrophages, suggesting that Kim1 overexpression may increase local recruitment and/or expansion of renal macrophages. The close physical association between F4/80^+^ cells and Sox9^+^/Kim1^+^ PTECs in PTEC DN RAR kidneys supports this finding and suggests that these cells are recruiting and/or causing expansion of renal macrophages locally. This is also consistent with our finding that there are increased numbers of proliferating, EdU^+^ F4/80^hi^ renal macrophages in PTEC DN RAR mice after injury. However, unlike the conditional Kim1 overexpression studies mentioned above (30), our data show that this increase in renal macrophages is associated with suppression of inflammatory cell activation in uninjured and injured PTEC DN RAR mouse kidneys after IRI-AKI, perhaps explaining why renal macrophage recruitment by Kim1 upregulation in PTEC DN RAR mice does not promote renal injury.

Kim1 is expressed by dedifferentiated and proliferating PTECs after AKI (56, 57), but in PTEC DN RAR mice, Kim1 expression is increased after AKI, despite there being a reduction in tubular injury. This is associated with reduced LTL staining, a marker of differentiated PTECs; increased expression of Sox9, a transcription factor that is expressed by dedifferentiated PTECs (58–60); and increased expression of Ki67, a marker of cell proliferation. There is also increased expression of Vimentin, another marker of PTEC dedifferentiation (19), in Kim1^+^ clusters of cells in uninjured PTEC DN RAR mice. This suggests that generalized activation of PTEC dedifferentiation results in increased Kim1 expression in PTEC DN RAR mouse kidneys. An alternative explanation is that improved PTEC health (e.g., due to increased Sox9 expression and/or glycolytic flux as discussed above) increases Kim1 expression by increasing protein synthetic function of injured PTECs. While this remains a possibility, the fact that there is widespread expression of Sox9 in uninjured PTEC DN RAR mice and increased glycolytic flux, a metabolic feature of dedifferentiated and proliferating PTECs (90, 91), in cultured PTECs from PTEC DN RAR mice suggests that increased Kim1 expression results from a more generalized effect on PTEC differentiation. This is also consistent with the known effects of RA in promoting cellular differentiation in a variety of contexts (35, 92), including glomerular podocyte differentiation after injury (36, 37). In addition, our findings are consistent with data showing that RA inhibits glycolysis in a variety of cultured cell types (41). However, decreased glycolysis is associated with RA-induced cellular differentiation in many of these studies, so it is unclear whether the effects we observe in DN RAR PTECs on glycolytic flux are direct or are secondary to dedifferentiation of these PTECs.

Our analysis of RARE-LacZ reporter mice indicates that RAR activation in PTECs is heterogeneous. We speculate that RAR signaling is activated in PTECs exposed to different levels of injury and that this exerts different effects on PTEC differentiation and Kim1 expression depending on the severity of injury. Activation of RAR signaling in PTECs with less severe injury may be sufficient to inhibit dedifferentiation and thereby prevent injury-induced Kim1 expression. Likewise, sporadic PTEC RAR activation may occur in response to minor injuries resulting from everyday fluctuations in renal blood flow/hydration, and this may be sufficient to prevent spontaneous dedifferentiation of PTECs in uninjured mice. Conversely, activation of RAR signaling in more severely injured PTECs may not prevent the induction of a dedifferentiation response associated with expression of Kim1. In these contexts, RAR signaling may be acting in a cell-autonomous fashion or, perhaps more likely, in an indirect, paracrine fashion so that localized activation of RAR signaling in a small number of PTECs could have widespread effects on PTEC dedifferentiation throughout the tubular segment.

In summary, our studies suggest that the protective effect of inhibiting RAR signaling in PTECs is mediated by increased Kim1-dependent efferocytosis, and we show that this increase in Kim1-dependent efferocytosis is associated with increased dedifferentiation, proliferation, and metabolic reprogramming of PTECs. We therefore propose that activation of RAR signaling in the kidney after AKI plays a role in preserving renal function, thus ensuring animal survival by reducing dedifferentiation and thereby preventing further loss of function of surviving PTECs from regions of the kidney with less severe tubular damage after a renal insult.

## Methods

### RNA-Seq studies

Differentially expressed gene lists comparing human control and AKI kidney sample bulk and snRNA-Seq were obtained from Supplemental Table 2 in Hinze et al. (see Supplemental Methods for details about patient and control samples) (49). RNA isolation, quality control, and bulk RNA-Seq for the mouse CD11B cell RNA-Seq studies were performed as described in the Supplemental Methods. GSEA were performed using the clusterProlifer R package (93) using GO classification from the MSigDB collections (94, 95), and macrophage and RA target gene sets from published data (50, 66, 67), as described in the text.

### Mouse mutant lines and strains

Male BALB/c mice were purchased from Charles River Laboratories. *RARE-hsp68-LacZ* (referred to as RARE-LacZ mice in the text) (on a CD-1 background) were purchased from The Jackson Laboratory (51). *PEPCK-Cre* mice on a mixed 129svJ; C57BL/6 background were from Volker Haase (Division of Nephrology, Vanderbilt University Medical Center, Nashville, Tennessee, USA; ref. 53), and *R26R-DN RAR* on a C57BL/6 background were from Cathy Mendelsohn (Department of Pathology, Columbia University, New York, New York, USA; ref. 33). Mice were genotyped and identified from ear-punch biopsies using allele-specific PCR primers, and copy number variant (CNV) genotyping was performed to identify homozygous *R26R-DN RAR* mice (see Supplemental Methods). Genotyping primers are listed in Supplemental Table 5A.

#### AKI models.

Numbers were randomly assigned to each mouse and split equally into groups for each study. Another individual performed assays and data analyses and was blinded to group assignments until analyses were completed. Rhabdo-AKI was induced in 13- to 14-week-old male mice with intramuscular glycerol, unilateral IRI-AKI with delayed nephrectomy (IRI-AKI DN) was performed on 10- to 12-week-old male mice, and tGFR were measured at day 27; each procedure was as previously described (52, 55, 96). Mice that underwent right nephrectomy alone were controls. For bilateral IRI-AKI, 10- to 12-week-old male mice underwent bilateral renal pedicle clamping for 28 minutes. For bafilomycin A1 studies, mice were injected IP with 3 mg/kg bafilomycin A1 (Med Chem Express LLC, 50-196-7747) in corn oil 1 hour before IRI-AKI surgery, as described (27). Controls were given corn oil. Detailed information about AKI models and renal function assays is provided in Supplemental Methods.

### Tissue analyses

Kidneys were collected after terminal cardiac perfusion for snap freezing (for RNA) and preparation of formalin fixed, frozen (FFF) and formalin fixed, paraffin embedded (FFPE) tissue blocks. Histological scoring for fibrosis and tubular injury was performed by observers blinded to the experiment, as described (52, 97) (see Supplemental Methods for details).

#### β-Gal and IF analysis.

β-Gal staining and antibody colabeling was performed on FFF sections, as described (41). IF and TUNEL staining were performed on FFPE or FFF sections, as described in Supplemental Methods. Lectins, primary and secondary antibodies, dilutions, biotin amplification, and antigen retrieval methods are described in Supplemental Table 5, B and C. Digital images were scanned using Zeiss AxioScan Z1 slide scanner (Carl Zeiss Microscopy GmbH, 10×), and downloaded into QuPath (version 0.4.3). Kidney regions were identified using established landmarks. Images were quantified as surface area stained in the indicated regions or the ratio of cells staining with the respective antibody, using DAPI staining to quantify cell nuclei using QuPath. Cell numbers and types were identified based on average values of fluorescence threshold detection using machine learning classifiers in QuPath (see Supplemental Methods).

#### RNA extraction and quantitative PCR.

RNA purification and quantitative PCR (qPCR) was performed using iQ SYBR Green super mix (Bio-Rad) using a Bio-Rad CFX96 real-time PCR system, as described in Supplemental Methods. Primers pairs were obtained from PrimerBank or designed using the National Center for Biotechnology Information Primer Designing tool (97). PrimerBlast was used to confirm that primers spanned exon junctions. Primer sequences are listed Supplemental Table 5D.

### Isolation and characterization of cultured PTECs

PTECs were enriched from homogenized mouse kidneys using LTL-conjugated microbeads, cultured, and underwent IF staining, as described in Supplemental Methods. Cells were used between passages 2 and 3. Antibodies used to characterize PTECs are indicated in Supplemental Table 5, B and C. To evaluate cell growth, P2 PTECs were seeded in triplicate onto a 96-well plate at a density of 7.5 × 10^3^ cells per well. Wells were coated with collagen 1, and cells were grown in complete PTECs media. Cells were counted after 6 hours (time [t] = 0), 24 hours, and 72 hours. Wells were washed in PBS, fixed with 100% methanol, and stained with DAPI, and nuclear counts were determined from digital images acquired from 2 randomly selected 10× objective fields per well (6 fields/mouse) at each of the indicated time points.

#### Seahorse assays.

Extracellular acidification rates (ECAR, XF Glycolytic Stress Test) and oxygen consumption rates (OCR, XF Cell Mito Stress Test) were performed with a Seahorse XF24 Extracellular Flux Analyzer and test kits (Agilent Technologies Inc.), as outlined in Supplemental Methods. The ECAR (mpH/min) and OCR (pMoles O_2_/min) was measured in real time, and glycolytic and mitochondrial parameters were calculated using Wave software (Agilent). After the assays, cells were fixed with 100% methanol and stained with DAPI. Values were normalized for count of DAPI^+^ nuclei in each well.

#### Endocytosis of oxidized LDL.

Cells were plated onto 8-well chamber slides (iBidi, 80826) at a density of 1 × 10^5^cells/well. After the cells became approximately 70 % confluent, media were changed to serum-free DMEM media to starve the cells for 6 hours. Dil-OxLDL (20 μg/mL, Thermo Fisher Scientific, L34358) was added into the media for 2 hours, incubated at 37°C. Cells were then washed 3 times, fixed with 5% Formalin-PBS, and costained with Kim1 antibody.

### Flow cytometry

Kidneys were homogenized and prepared for fluorescence antibody staining, as described in Supplemental Methods. Antibodies, dilutions, and fluorophores used are in Supplemental Table 5E. For EdU detection, 1 mg/mouse EdU (Invitrogen, C10424) was given i.p. 2 hours before sacrifice. After antibody staining, cell fixation, and permeabilization, cells were resuspended in Click-iT Plus cocktail solution and incubated for 30 minutes on ice. Fluorescence-minus-one (FMO) controls were prepared for each cell marker. Data were collected on a LSR II (BD Biosciences) equipped with 5 aligned 355, 405, 488, 532, and 633 nm lasers. Data analysis, including sequential gating based on FMO controls, was performed by using BD FACS diva software.

### Statistics

Statistical analyses were performed using GraphPad Prism 9.3 software, and results are expressed as means ± SEM, with individual data points shown where indicated. Two-tailed, unpaired *t* tests were used to compare 2 groups, 1-way ANOVA was used to compare between 3 or more groups, and 2-way ANOVA was used to compare groups over time. If *P* < 0.05 for between-group analyses, multiple between-group comparisons were performed controlling for FDR of *P* < 0.05 using the 2-step method of Benjamini, Krieger, and Yekutieli. The *q* values are indicated after correction for multiple between-group comparisons. Mantel-Cox log-rank test was used to compare survival between groups. Because of the large numbers of mice in the PTEC DN RAR studies, we randomly selected subsets of samples for analysis of histology, IF, and RNA studies before performing the assays.

### Study approval

All mouse experiments were approved by the Vanderbilt IACUC.

### Data availability

All relevant data are found in this article and in the Supporting Data Values file. CD11B cell bulk RNA-Seq data were deposited with the NCBI Gene Expression Omnibus repository (GSE239850, GSE239850). Human RNA-Seq data used for GSEA analysis were obtained from differentially expressed gene lists in Supplemental Table 2 and UMAP plots of the snRNA-Seq data sets (https://shiny.mdc-berlin.de/humAKI/) from Hinze et al. (11).

## Author contributions

MY designed, conducted, and analyzed experiments, with assistance from LNL, MB, KC, YZ, RD, and AM, who helped conduct and analyze experiments. HY performed tubular injury scoring. TV and AJD assisted with data analysis (GSEA studies). RCH, LG, and CRB helped design studies and interpret data. RCH, LG, CRB, and AJD edited the manuscript and figures. MDC conceptualized and planned studies, interpreted data, prepared figures, and wrote the manuscript.

## Supplementary Material

Supplemental data

Supplemental table 1

Supplemental table 2

Supplemental table 3

Supplemental table 4

Supplemental table 5

Supporting data values

## Figures and Tables

**Table 1:**
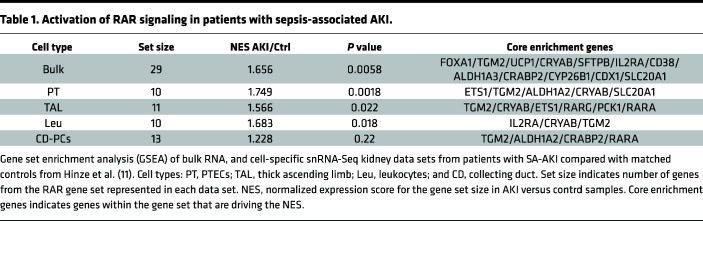
Activation of RAR signaling in patients with sepsis-associated AKI.

**Table 2:**
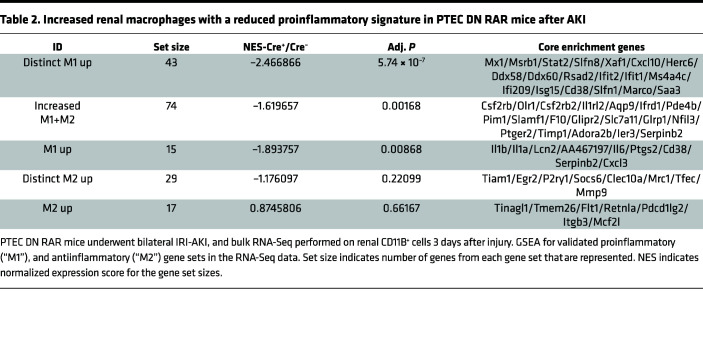
Increased renal macrophages with a reduced proinflammatory signature in PTEC DN RAR mice after AKI

## Version 1 09/12/2023

In-Press Preview

## Version 2 10/23/2023

Electronic publication
