# Supplementary material for: Inhibition of retinoic acid signaling in proximal tubular epithelial cells protects against acute kidney injury
Source: JCI Insight. 2023 Oct 23;8(20):e173144. doi: 10.1172/jci.insight.173144 (PMC10619506; doi:10.1172/jci.insight.173144)
Supplement: Supplemental data [file jciinsight-8-173144-s247.pdf]

# S. Figure 1

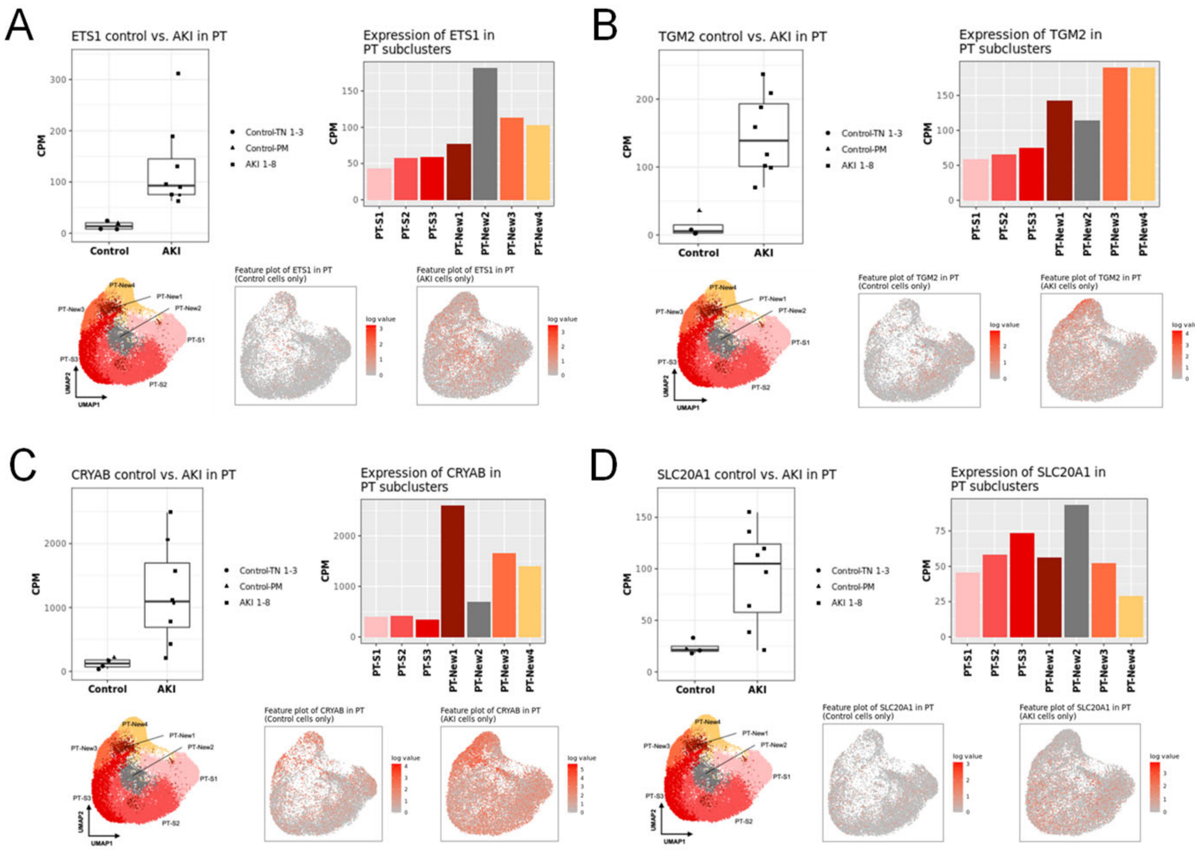


E

| Symbol | AKI/Control | pvalue | padj |
|---|---|---|---|
| HAVCR1 | 4.239929274 | 4.0748E-07 | 1.80172E-05 |

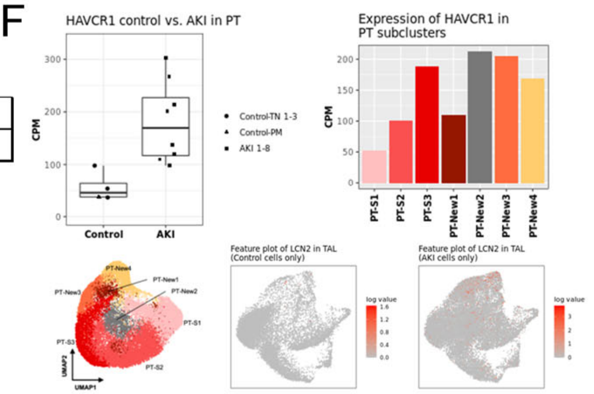


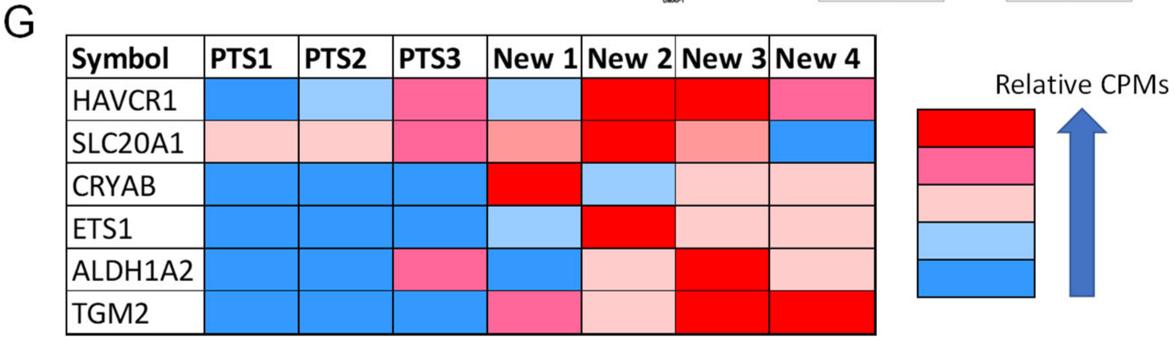


**S. Figure 1. Expression of RAR target genes in proximal tubular epithelial cells from patients with SA-AKI.** A-D, Expression of RAR core enrichment genes identified from GSEA analysis of the PTEC snRNA seq dataset from Hinze et al. 2022. E/F, Expression of Kim-1/Havcr1. Figures were generated using on-line software developed for these datasets at https://shiny.mdc-berlin.de/humAKI/. Gene expression in controls vs. AKI patients in reads per million. UPMAP plots indicate PTEC subclusters identified from snRNA seq studies. Feature plots showing distribution of genes in PTECs clusters summarized in bar charts. G, Visual representation of the distribution of RAR target genes in PTEC clusters.

# S. Figure 2

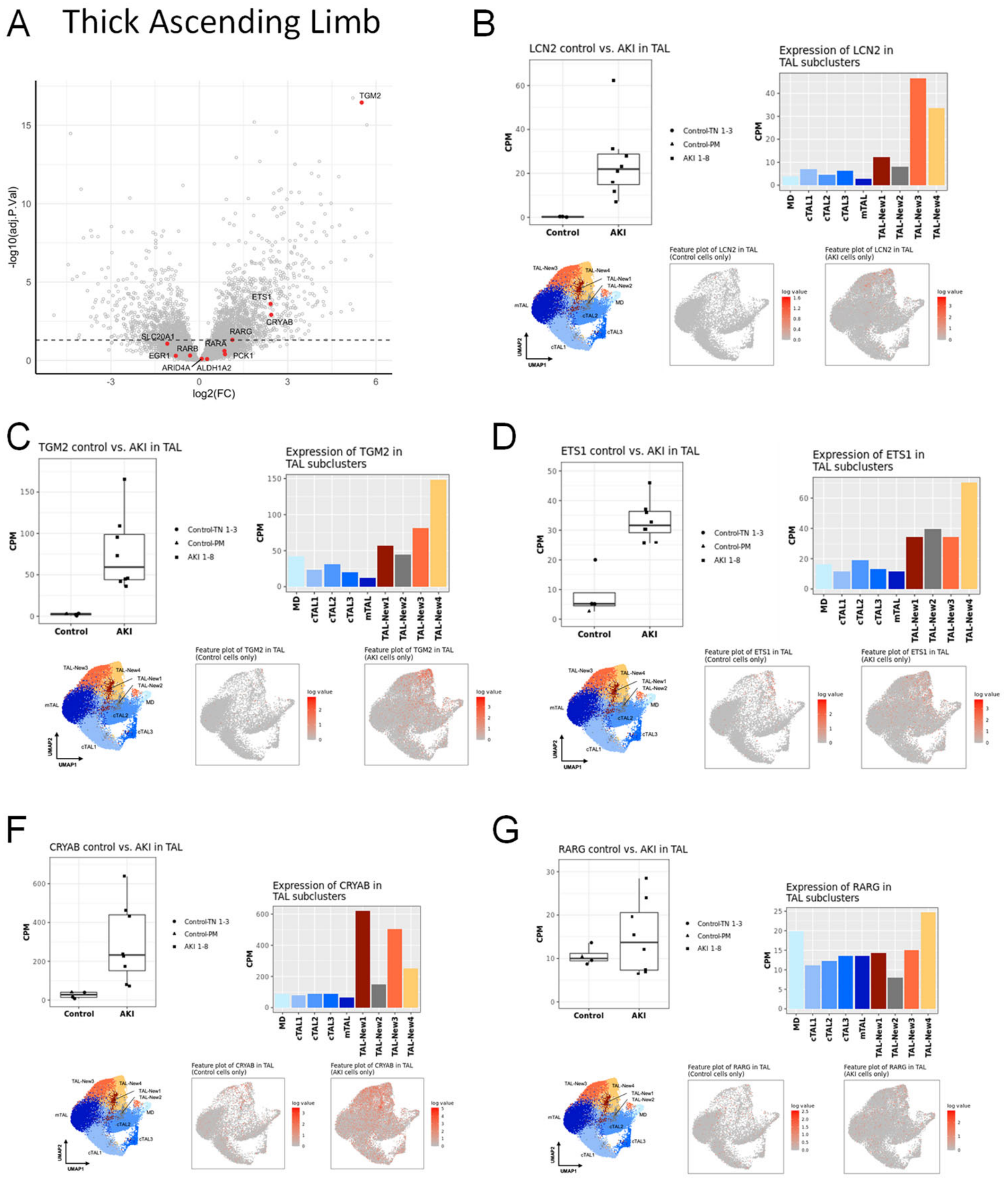


**S. Figure 2. Expression of RAR target genes in thick ascending limb cells from patients with SA-AKI. A,** Volcano plot indicating fold change in expression of core enrichment gene (AKI vs. controls) from the TAL snRNA seq dataset from Hinze et al. 2022. Dotted line indicates p<0.05E. B, Expression of N-Gal/Lcn2. C-G, Expression of RAR core enrichment genes identified from GSEA analysis of the TAL snRNA seq dataset from Hinze et al. 2022.[1] Figures were generated using on-line software developed for these datasets at https://shiny.mdc-berlin.de/humAKI/. Gene expression in controls vs. AKI patients in reads per million. UPMAP plots indicate PTEC subclusters identified from snRNA seq studies. Feature plots showing distribution of genes in TAL subclusters summarized in bar charts.

# S. Figure 3

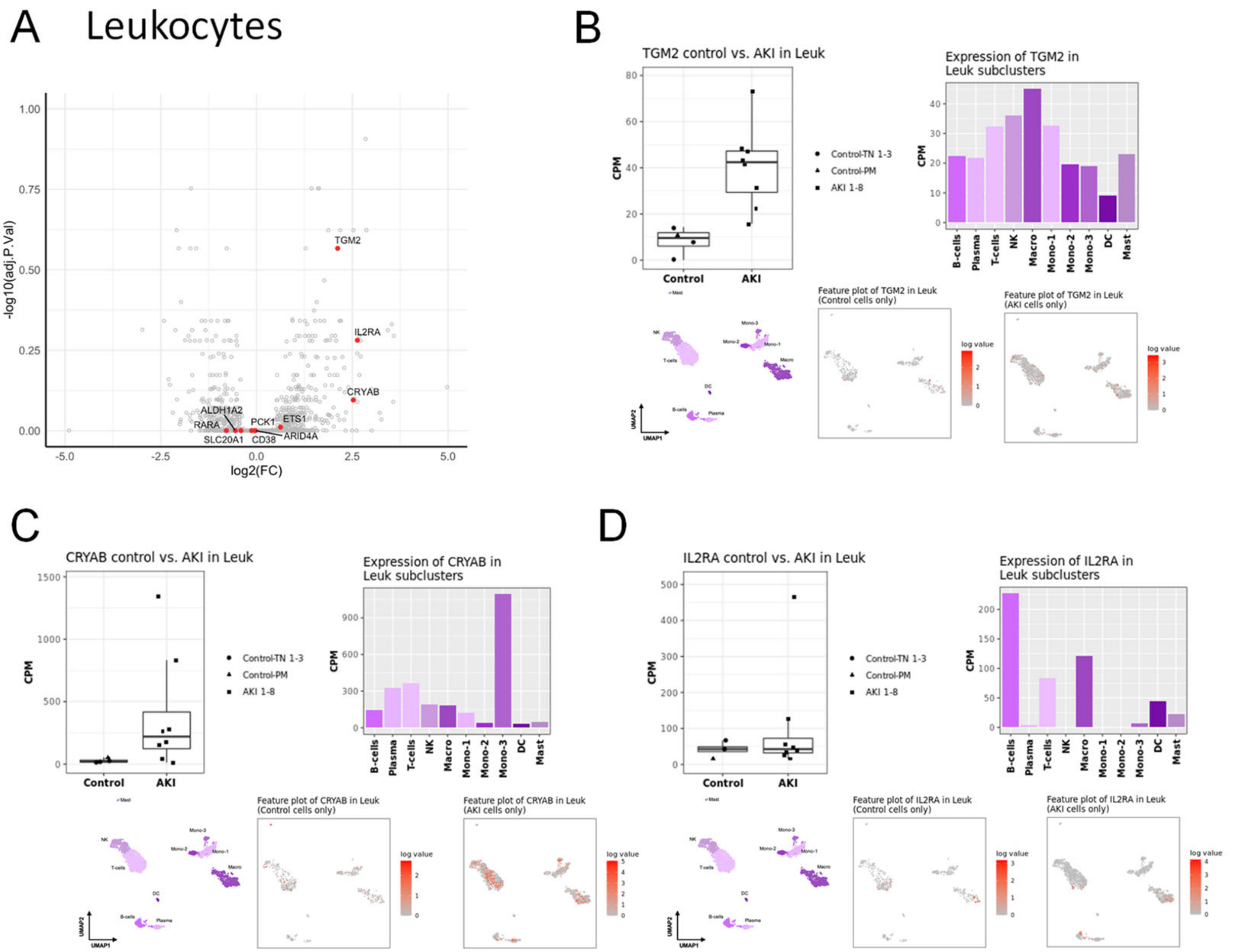


S. Figure 3. Expression of RAR target genes in renal leucocytes from patients with SA-AKI. A, Volcano plot indicating fold change in expression of core enrichment gene (AKI vs. controls) from Leukocyte snRNA seq dataset. Dotted line indicates p<0.05E. B-D, Expression of RAR core enrichment genes identified from GSEA analysis of the Leu snRNA seq dataset from Hinze et al. 2022. Figures were generated using on-line software [https://shiny.mdc-berlin.de/humAKI/](https://shiny.mdc-berlin.de/humAKI/). Gene expression in controls vs. AKI patients in reads per million. UPMAP plots indicate leukocyte subsets identified from snRNA seq studies. Feature plots showing distribution of genes in leukocyte subsets summarized in bar charts.

# S. Figure 4

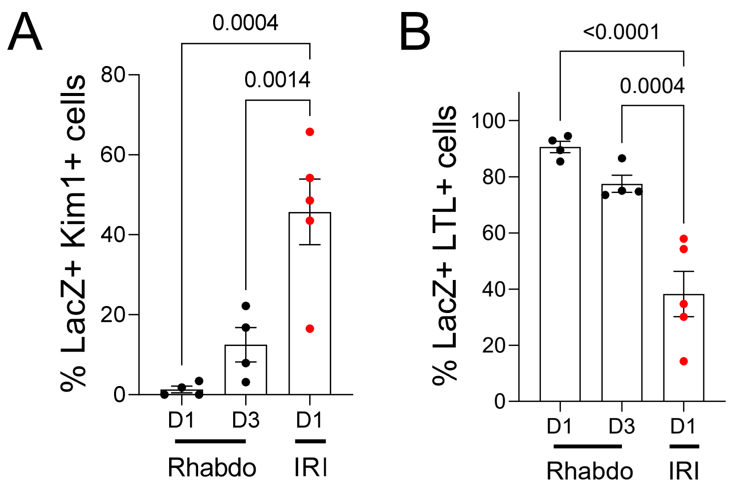


**S. Figure 4. Increased activation of RAR signaling in injured, Kim1 positive PTECs after IRI- compared with rhabdo-AKI.** RARE-LacZ reporter mouse kidneys were used to evaluate the cellular localization of LacZ staining after IRI- and Rhabdo-AKI. IRI-AKI imaging data obtained from Chiba et al. 2016. [2] After LacZ staining, kidney sections were stained with LTL and antibodies to Kim1. LTL, Kim1 and LacZ staining evaluated in the OSOM. A, The % LacZ positive $Kim1^+$ cells (injured PTECs); B, The % LacZ positive $LTL^+$ $Kim1^-$ cells (uninjured PTECs) at Day 1 and 3 after rhabdo-AKI, and Day 1 after IRI-AKI (maximal RARE-LacZ activation after IRI-AKI[2]). Results expressed as mean +/- SEM, with individual data points shown. 1 way ANOVA used to compare between groups, q values shown for between group comparisons after correcting for repeated testing. Note: the Day 1 and 3 Rhabdo-AKI data are the same as the time course line graphs in the longer-term time course studies represented in Fig. 3A and C.

# S. Figure 5

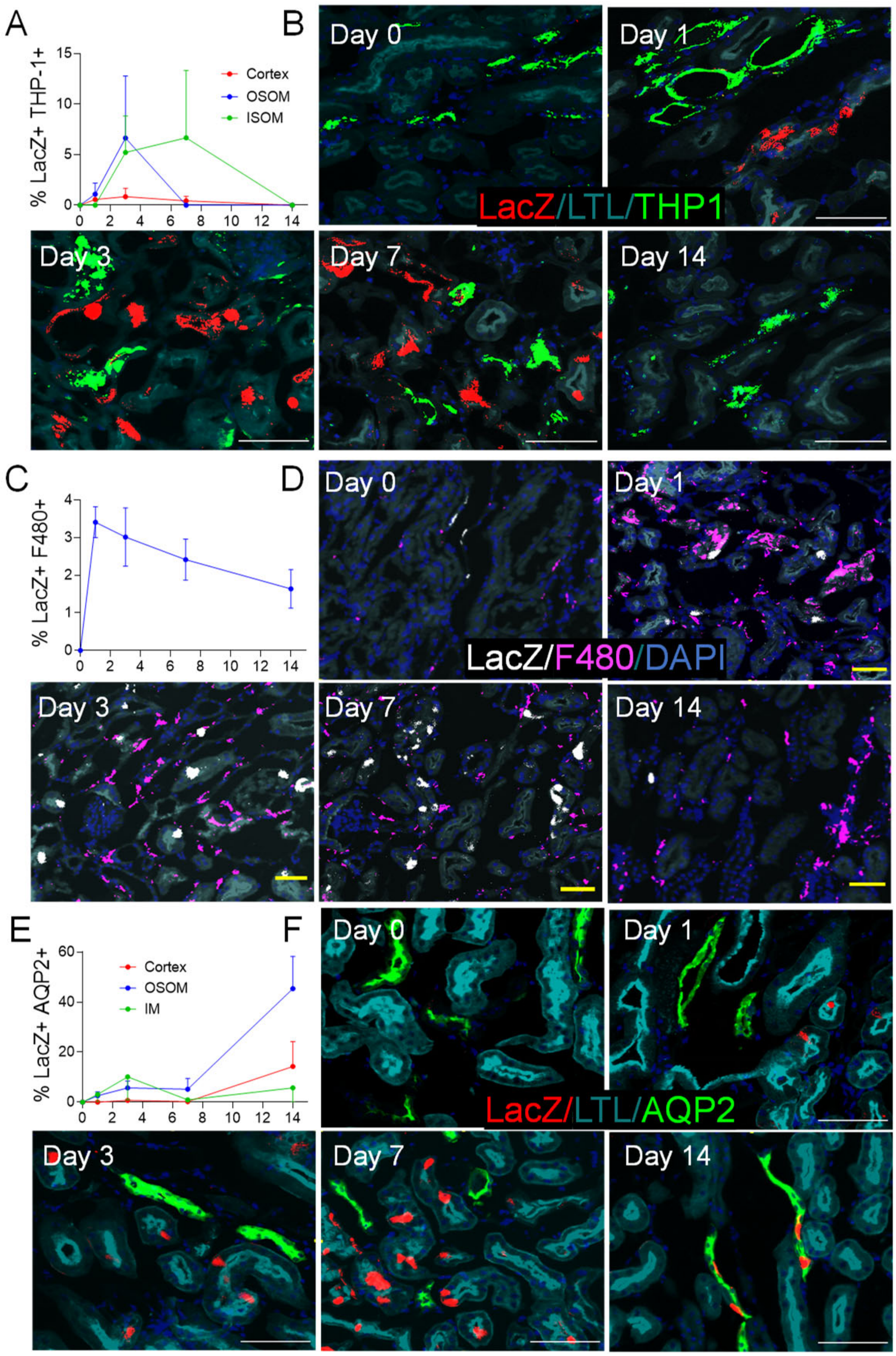


**S. Figure 5. RAR signaling in thick ascending limb, renal macrophages, and collecting ducts after rhabdo-AKI.** RARE-LacZ reporter mice were used to evaluate the cellular localization of RAR signaling after rhabdo-AKI. After LacZ staining, kidney sections were stained with LTL, Tam Horsfall Protein -1 (THP-1, thick ascending limb (TAL)), F4/80 antibodies (renal macrophages), and AQP2 (collecting duct (CD), principal cells). A/B, Activation of RAR signaling in TAL. The % LacZ positive THP-1$^+$ TAL cells at different time points in the cortex, OSOM, and ISOM (A). Representative images showing LacZ, THP-1 and LTL staining in the OSOM (B). C/D, Activation of RAR signaling in renal macrophages. The % LacZ positive F4/80$^+$ renal macrophages in the cortex and OSOM (C). Images showing LacZ, F4/80, and LTL staining in the OSOM (D). E, Activation of RAR signaling in CDs. The % LacZ positive AQP2$^+$ CD cells at different time points after injury in the cortex, OSOM and IM (E). Images from the OSOM showing LacZ, AQP2, and LTL staining in the OSOM (F). Graphical results expressed as means +/- SEM A/E, from 2 mice before injury (Day 0), 4 mice at Days 1 and 3; 3 at Day 7; and 7 at Day 14 after injury. C, 2 mice Day 0, 5 mice at Days 1 and 3; 3 at Day 7; and 3 at Day 14 after injury. Scale bars B/F: 100μM; D: 50μM.

# S. Figure 6

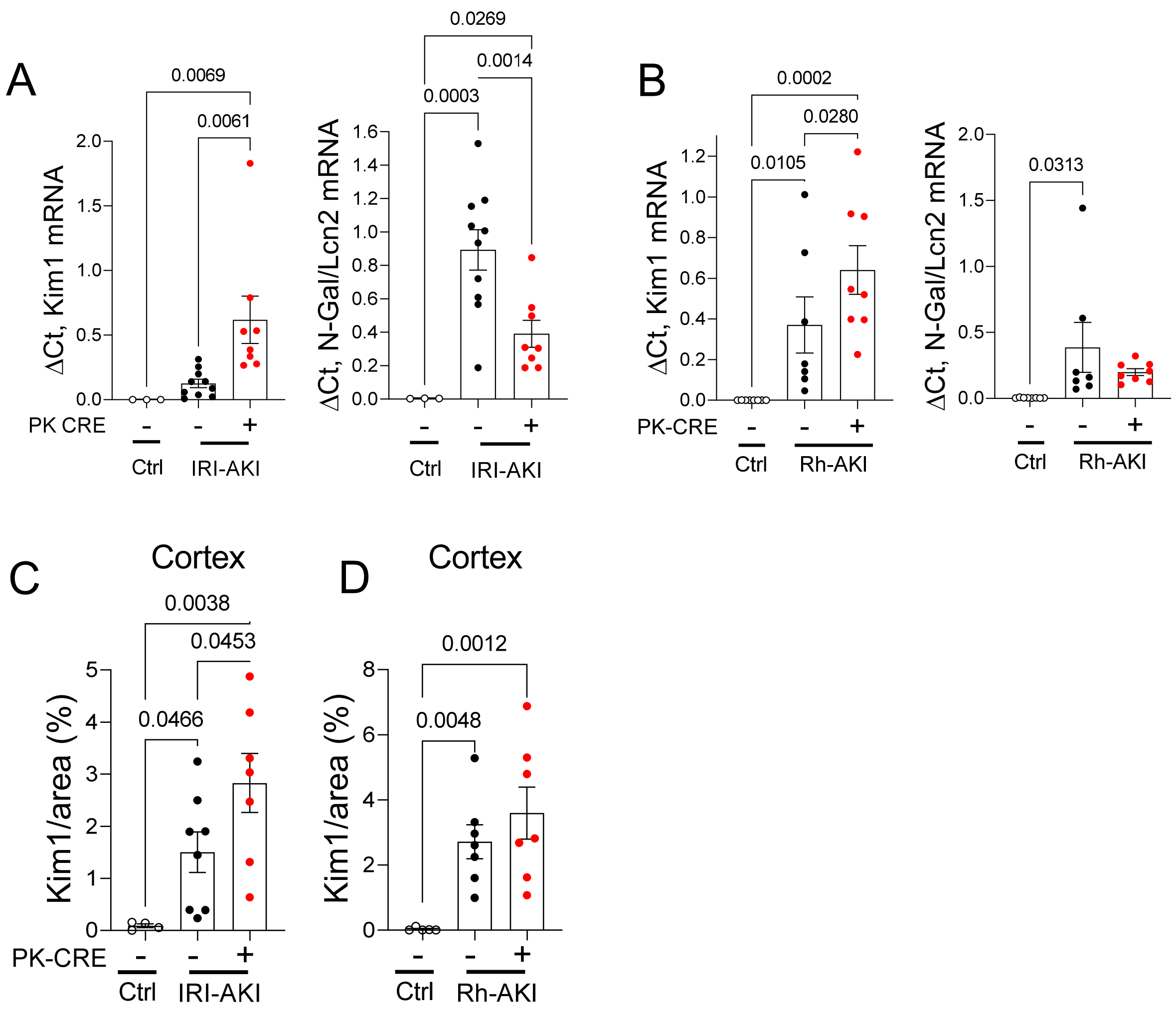


**S. Figure 6. Expression of injury markers Kim-1 and N-Gal in the PTEC DN-RAR kidneys after IRI- and Rhabdomyolysis-AKI.** PTEC DN-RAR mice underwent bilateral IRI-AKI, or rhabdo-AKI (Rh-AKI), and kidney harvested after 3 days. **A/B,** Renal *Kim-1* and *N-Gal* mRNAs by quantitative RT-PCR (QRT-PCR) after bilateral IRI-AKI, and rhabdomyolysis-AKI. **C,** Quantification of Kim-1 immunostaining as the % surface area in the cortex after IRI- and Rhabdo-AKI. Results expressed as means+/-SEM, with individual datapoints shown. 1-way ANOVA was used to compare between groups, and if p values <0.05, q values shown for between group comparisons after correcting for repeated testing.

# S. Figure 7

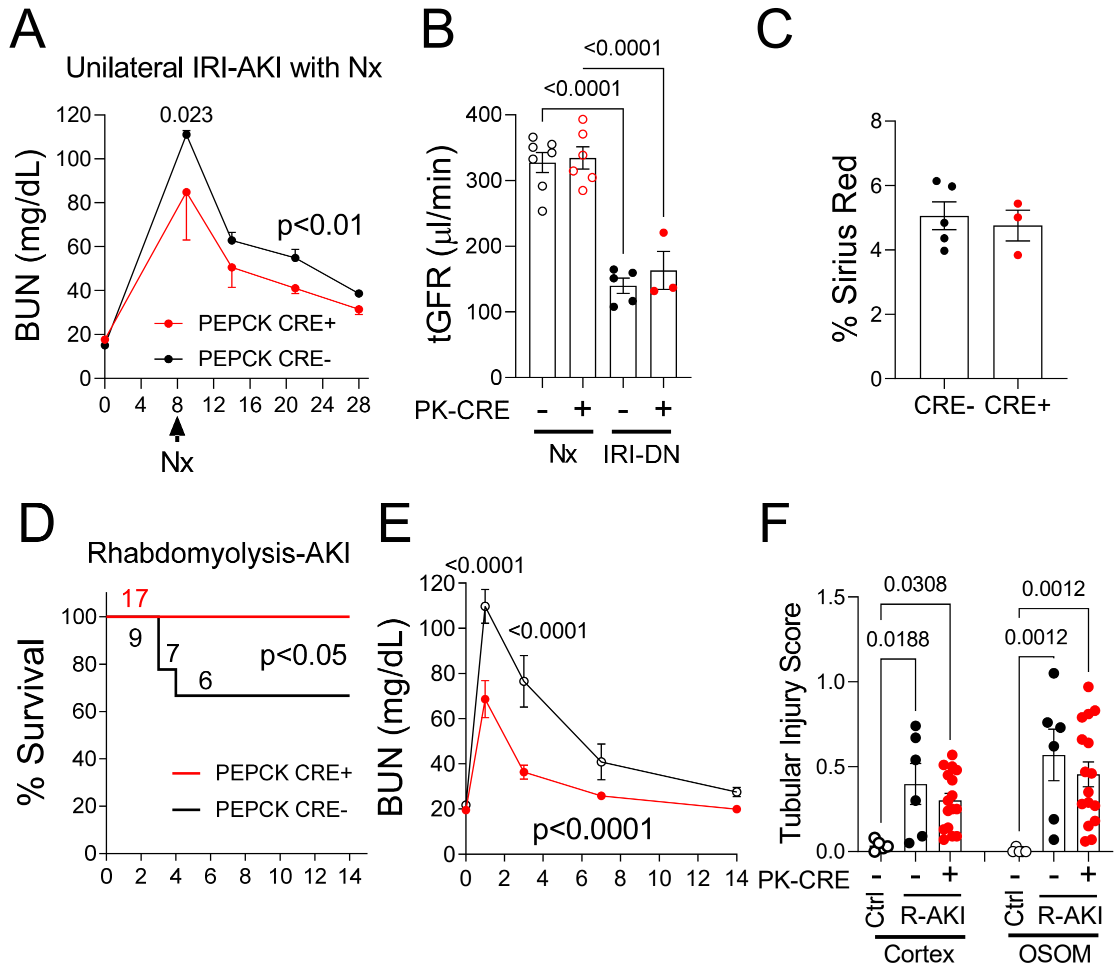


**S. Figure 7. Long-term studies show that inhibition of RAR signaling in PTECs protects against AKI at early time points after IRI- and rhabdo-AKI but does not improve long-term functional or histological recovery.** PTEC DN-RAR mice underwent unilateral renal pedicle clamping and delayed contralateral nephrectomy (Nx, unilateral IRI-AKI DN) and were followed up for 28 days after the initial injury, or rhabdomyolysis-AKI and were followed up for 14 days after injury. A-C, Unilateral IRI-AKI DN. A, BUN time course starting 1 days after Nx. Results expressed as means +/- SEM in mg/dl from 3 *PEPCK Cre+* and 5 *Cre-* controls. No mice died during these studies. B, Transdermal glomerular filtration rate (tGFR) corrected for body weight and expressed in µL/minute 26 days after injury. Results after injury (IRI-DN) compared with uninjured *Cre+* and *Cre-* controls 18 days after nephrectomy (Nx). Data points with mean +/- SEM are shown. C, Sirius red staining. Quantification as % surface area with Sirius red staining in the OSOM. D-F, Rhabdomyolysis-AKI. D, Survival curves. Mouse numbers indicated. E, BUN time course after injury, mouse numbers indicated in (D). F, Tubular injury scores. 14 days after injury in the cortex and OSOM in uninjured controls (Ctrl) and after rhabdo-AKI (R-AKI). A/E, 2-way ANOVA used to compare between groups over time. If p values <0.05 between groups, q values shown after correcting for repeated testing. B/C/F, 1-way ANOVA used to compare between groups, and if p values <0.05, q values shown for between group comparisons after correcting for repeated testing. D: Survival curves compared by Log-rank test, p<0.05.

S. Figure 8

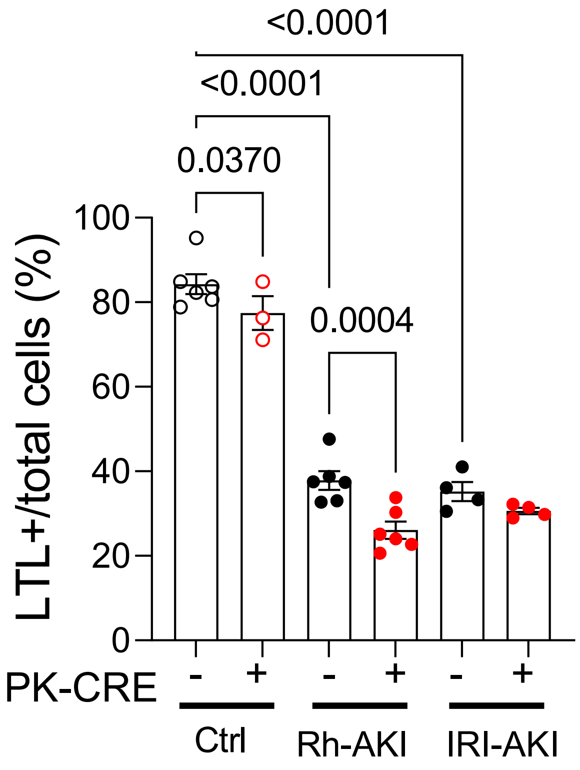


**S. Figure 8. LTL positive cells in PTEC DN-RAR mouse kidneys after Rhabdo- and IRI-AKI.** Quantification of LTL+ staining cells as a percentage of the total cell numbers (as determined by DAPI staining) in the OSOM of *PEPCK Cre+* and *Cre-* mouse controls (uninjured), and 3 days after Rhabdo- and IRI-AKI, as indicated. Representative images showing LTL staining are shown in Fig. 5B/D and G. Results expressed as means +/- SEM, datapoints shown. 1-way ANOVA used to compare between groups, and if p values <0.05, q values shown for between group comparisons after correcting for repeated testing.

# S. Figure 9

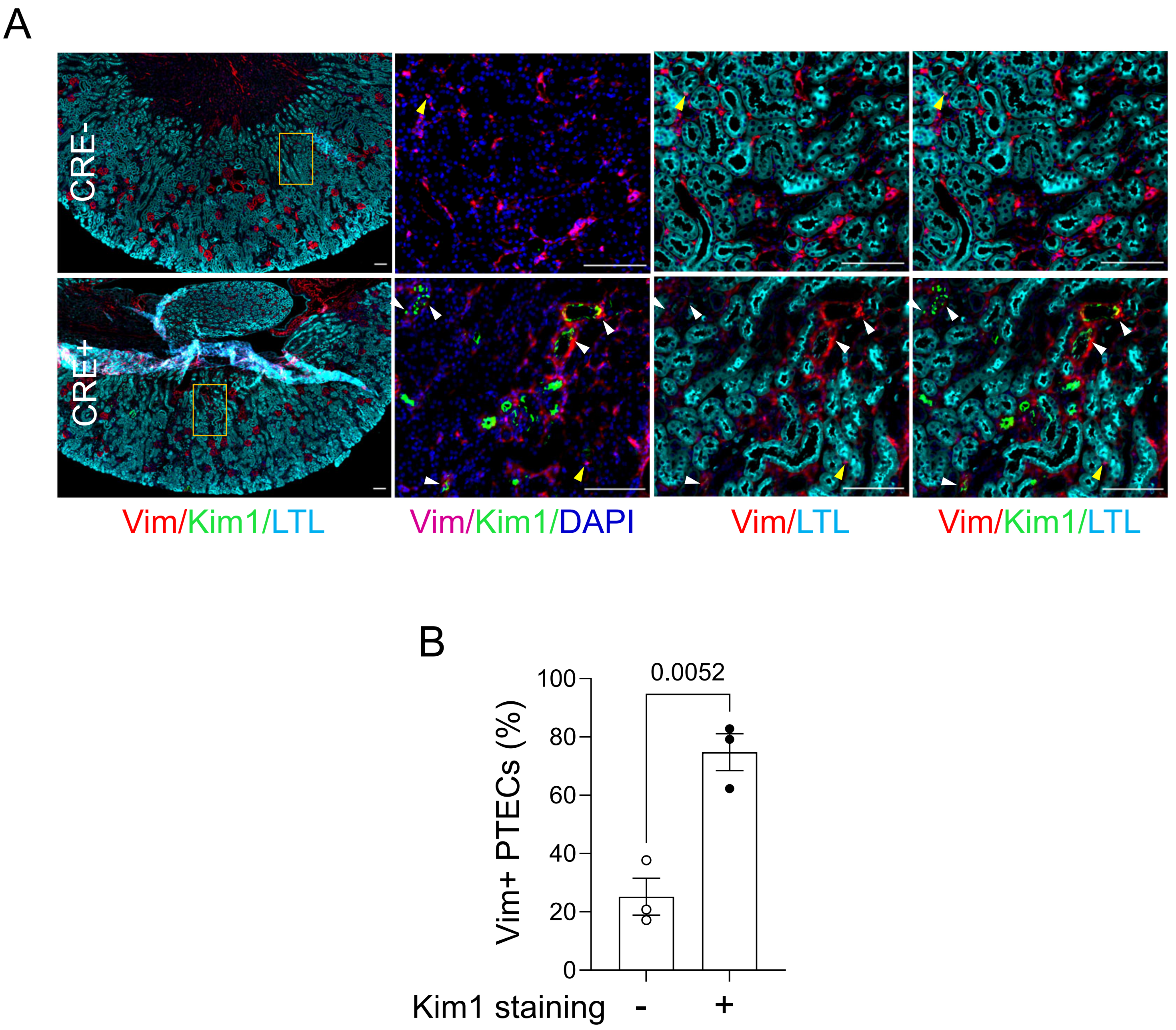


**S. Figure 9. PTEC expression of Vimentin and Kim1 in uninjured PTEC DN-RAR mouse kidneys.** A, Low and higher magnification images showing Vimentin (Vim), Kim1 and LTL staining in uninjured *PEPCK Cre+* and *Cre-* kidneys. Box insets in low magnification images indicate the regions used for higher magnification images. Yellow arrowheads indicate Vim1$^+$ LTL$^+$ Kim1$^-$ cells. White arrowheads indicate Vim$^+$ Kim1$^+$ cells. Scale bars=100µM. B, Quantification of the % Vim$^+$ cells that are LTL$^+$ Kim1$^-$ and Kim1$^+$ PTECs in the OSOM and cortex of uninjured *PEPCK Cre+* mice. We saw occasional Vim$^+$ Kim1$^-$ PTECs in Cre- mouse kidneys but no Kim1 staining, so only quantified Vim$^+$ Kim1$^{+/-}$ staining cells in Cre+ mouse kidneys.

# S. Figure 10

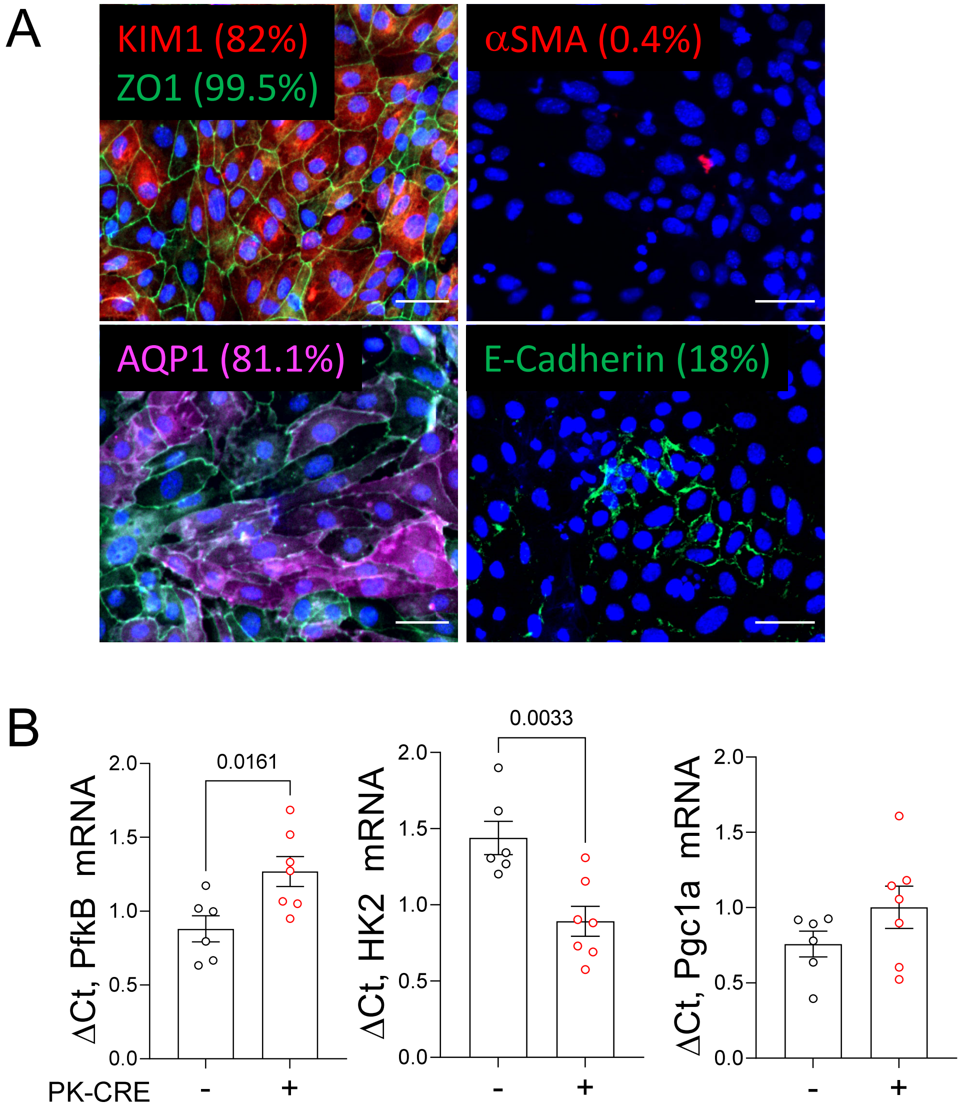


**S. Figure 10. Characterization of primary PTECs isolated from PTEC DN RAR mice.** Primary PTECs were isolated from homogenized and digested mouse kidneys and enriched using LTL-conjugated magnetic beads, as described in methods. **A,** Representative immunostaining of P2 PTECs Kim-1/ZO-1, AQP1, α-SMA, and E-Cadherin, with percentage of cells staining with the respective markers, as indicated. Scale bars=20μM. **B,** Quantification of *PfkB, Hk2,* and *Pgc1α* mRNAs by QRT-PCR in PTECs isolated from *PEPCK Cre+* and *Cre-* mice. Each data point is derived from RNA isolated from a separate P2 PTEC preparation from an individual mouse *Cre+* or *Cre-* mouse. Results are expressed as means +/- SEM, datapoints shown. T-tests were used to compare results in *PEPCK Cre+* and *Cre-* PTECs, p values shown if <0.05.

# S. Figure 11

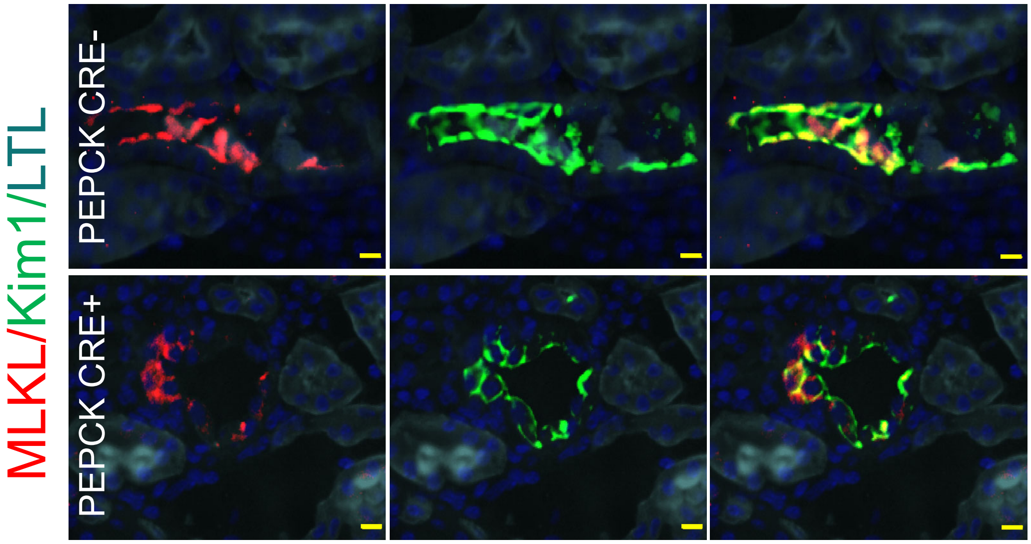


S. Figure 11. Subcellular localization of MLKL in *PEPCK Cre+* and *Cre-* mice after rhabdomyolysis-AKI. PTEC DN-RAR mice underwent rhabdo-AKI, and kidneys were harvested after 3 days. Representative images showing MLKL and Kim-1 staining with dominant membrane localization of MLKL in Kim-1$^{+}$ PTECs in *PEPCK Cre-* mice, and more diffuse cytoplasmic staining in Kim-1$^{+}$ PTECs in *PEPCK Cre+* mouse kidneys after Rhabdo-AKI. Scale bars=10μM

S. Figure 12

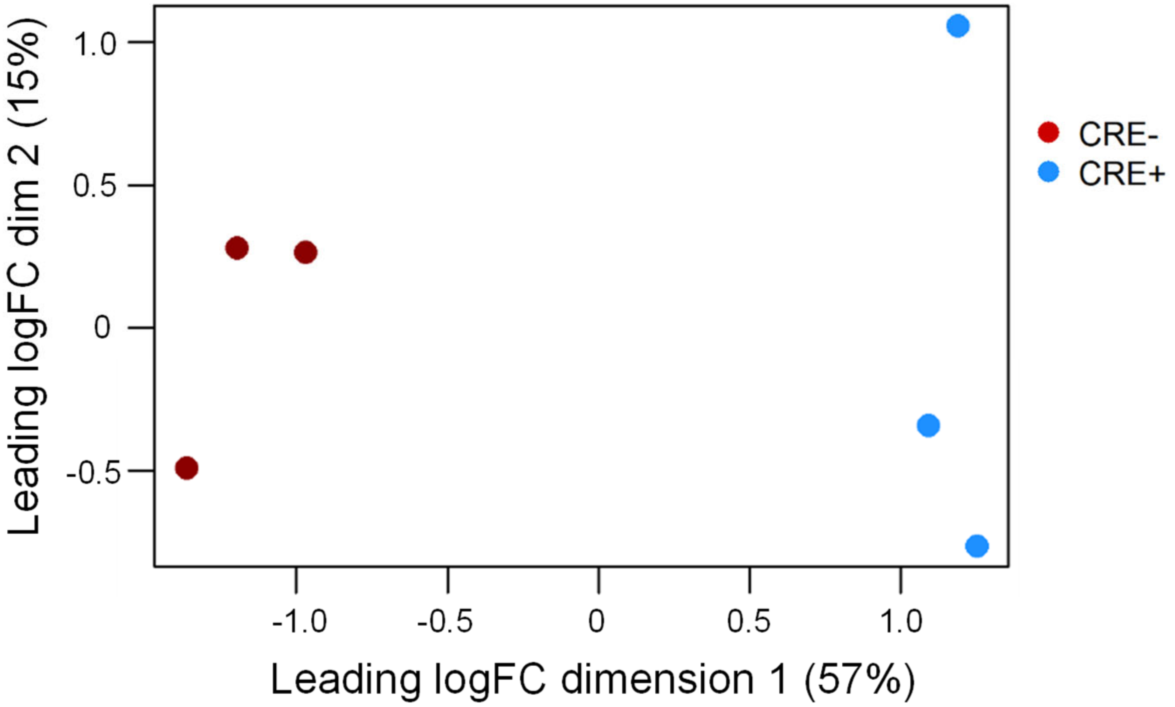


**S. Figure 12. Principal component analysis (PCA) of CD11B+ RNA seq data.** PTEC DN-RAR mice underwent bilateral IRI-AKI, and bulk RNA seq was performed on $CD11B^{+}$ cells from 3 *PEPCK Cre+* and 3 *Cre-* mouse kidneys 3 days after injury. PCA shows clear separation of *Cre+* and *Cre-* datasets in the first dimension, which accounts for 57% of the variation.

# S. Figure 13

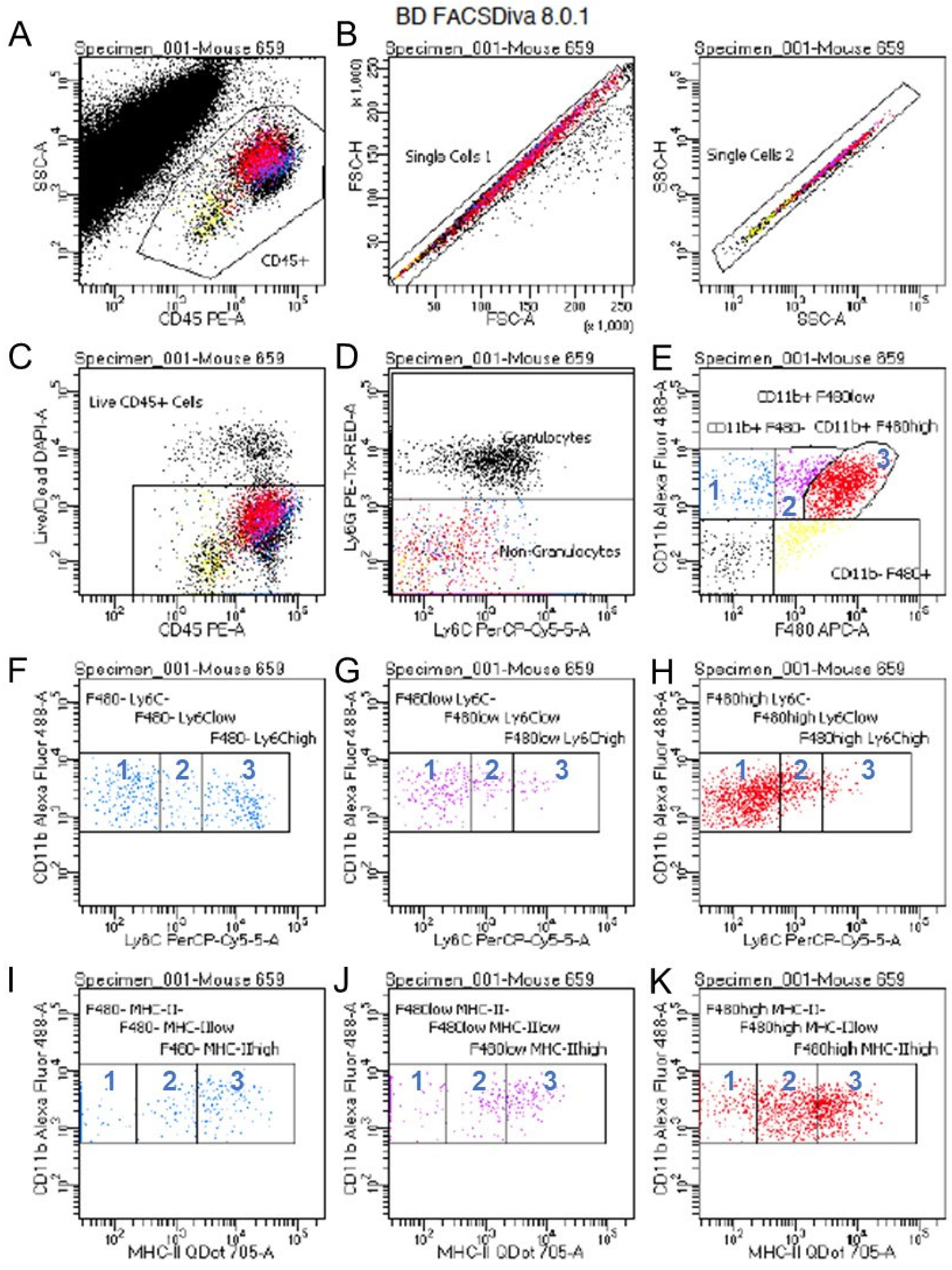


S. Figure 13. Flow cytometry gating strategy used to evaluate renal monocyte/macrophages. A, CD45$^+$ cells: CD45 vs SSC-A; B, CD45$^+$ single cells: FSC-H vs FSC-A, selected upper line followed by SSC-H vs. SSC-A select upper line; C, Single, CD45$^+$ live cells: DAPI$^-$ vs. CD45$^+$; D, Exclude CD45$^+$ granulocytes: Ly6G$^+$ (granulocytes) and Ly6G$^-$ cells (non-granulocytes); E, F4/80 expression in CD11B$^+$; Ly6G$^-$ cells: F4/80$^-$ (1); Intermediate (int), 2); high (hi) (3); F-H, Ly6C expression: F, F4/80$^-$: Ly6C$^-$ (1), Int (2), high (3); G, F4/80$^{Int}$: Ly6C$^-$ (1), Int (2), high (3); H, F4/80$^{Hi}$: Ly6C$^-$ (1), Int (2), high (3); I-K, MHC-II expression: I, F4/80$^-$: MHC$^{--}$ (1), Int (2), high (3); J, F4/80$^{Int}$: MHC-II$^-$ (1), Int (2), high (3); K, F4/80$^{Hi}$: MHC-II$^-$ (1), Int (2), high (3). Note: the flow plot shown in H is the same as that shown in Fig. 10F.

# S. Figure 14

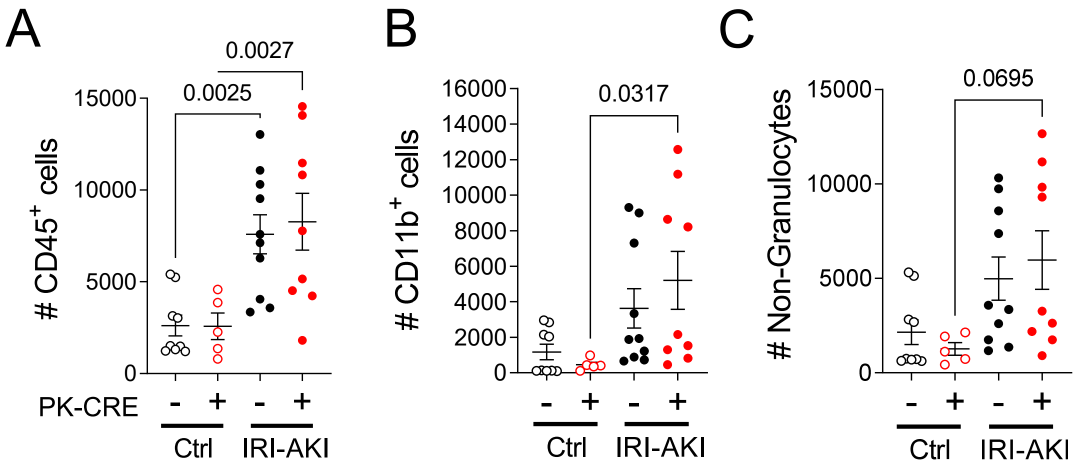


**S. Figure 14. Absolute numbers of renal CD45⁺ and CD11B⁺ cells in PTEC DN RAR mouse kidneys.** Kidneys were harvested from 5 *PEPCK Cre+* and 10 *Cre-* uninjured mice, and from 9 *PEPCK Cre+* and 10 *Cre-* mice 3 days after bilateral IRI-AKI. Tissue was digested and homogenized and evaluated by flow cytometry using antibodies and gating strategy illustrated in S. Fig. 11. A, Total numbers of live, single CD45+ cells acquired; B, CD11B+ cells; and C, non-granulocytes ($CD11B^+$; $Ly6G^-$). Results expressed as means +/- SEM, with individual data points shown. 1-way ANOVA was used to compare between groups, and if p <0.05, q values shown for between group comparisons, corrected for repeated testing.

# S. Figure 15

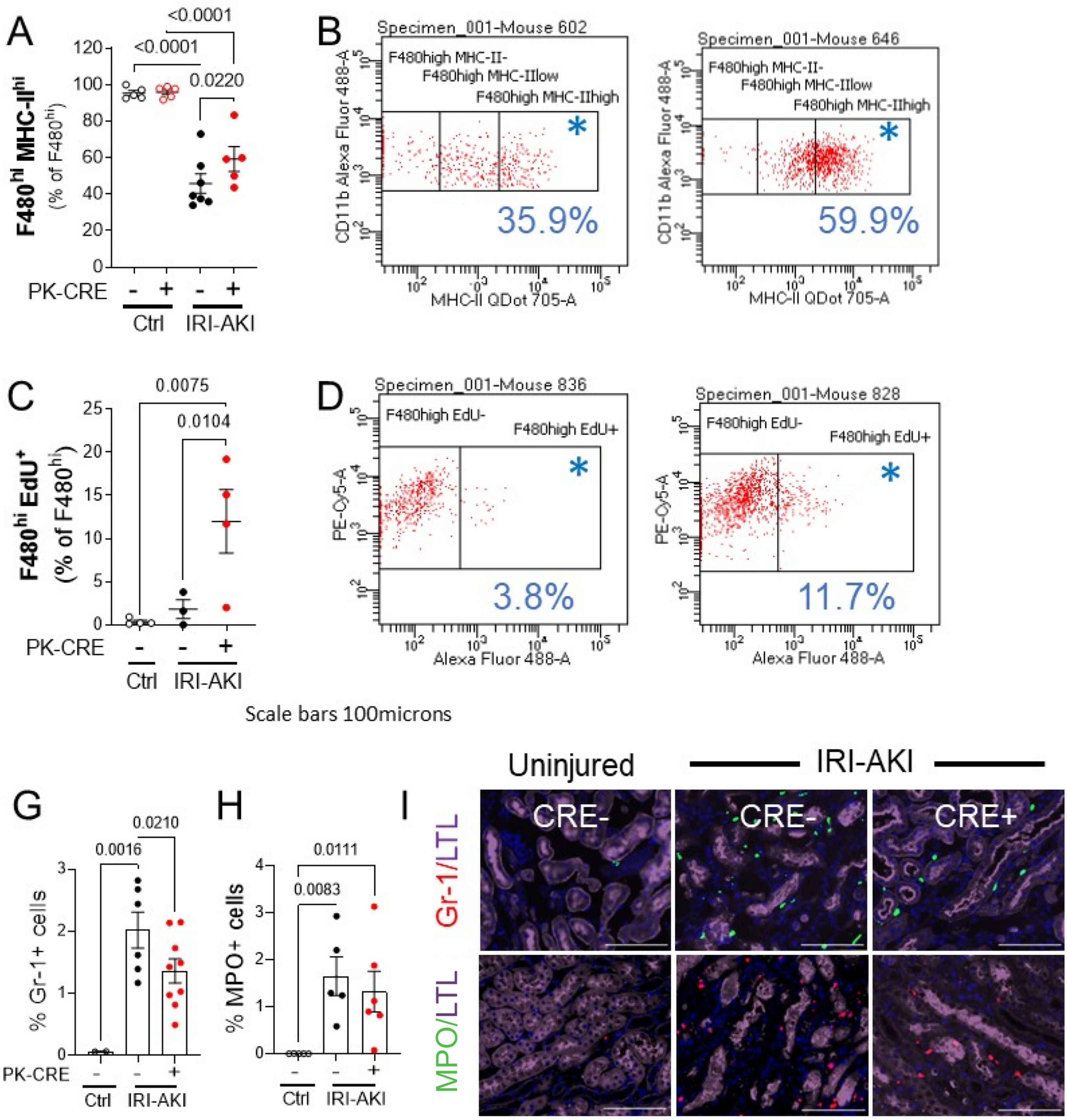


S. Figure 15. Flow cytometric and immunofluorescence analysis of renal monocyte/macrophages in PTEC DN RAR mice. Kidneys were harvested from PTEC DN RAR mice, uninjured and 3 days after bilateral IRI-AKI. Tissue was digested, homogenized, and evaluated by flow cytometry. A/B, MHC-II$^{hi}$ F4/80$^{hi}$ cells (kidney resident macrophages). A, MHC-II$^{hi}$ as the % of gated F4/80$^{hi}$ cells. B, Representative CD11B and MHC-II expression chart in *PEPCK Cre+* and *Cre-* mice after IRI-AKI (% in gated area indicated with *). C/D, EdU$^{+}$ F4/80$^{hi}$ cells (proliferating kidney resident macrophages). C, EdU labeled cells as the % of gated F4/80$^{hi}$ cells. D, Representative CD11B (Y axis) and EdU (X-axis) staining charts. E/F, Immunofluorescence staining with monocyte and neutrophil markers, Gr-1 and MPO. Kidneys were harvested from PTEC DN RAR mice 3 days after bilateral IRI-AKI and co-labeled with LTL and either Gr-1 (a marker of activated monocytes and neutrophils), or MPO (a marker of neutrophils) antibodies. G/H, G, Quantification of the Gr-1 and MPO+ cells as the % of the total cells counted. Results are expressed as means +/- SEM, with individual datapoints shown. 1-way ANOVA used to compare between groups, and if p <0.05, q values shown for between group comparisons, corrected for repeated testing.

## Supplemental Methods

RNA sequencing studies. Differentially expressed gene lists comparing human control and AKI kidney sample bulk and snRNA sequencing were obtained from S. Table S2 in Hinze et al. Genome Medicine 2022 (see S. Methods for details about patient and control samples) [1]. For mouse renal CD11B cell studies, whole kidneys were harvested after cardiac perfusion with 0.9% saline, kidneys were finely minced in DMEM with 0.3mg/ml Liberase DL (Sigma/Roche) and homogenized at 37°C in MACS C-tubes using the gentle MACS dissociator (Miltenyi Biotec), digestion neutralized with PEB buffer (2 mM EDTA, 1% BSA in PBS), and cell suspension passed through 40µm cell strainer. Cells were then incubated with anti-mouse CD11b MACS beads (Miltenyi Biotech, 130-126-725) for 15 minutes, and CD11b+ cells separated by loading cells into MACS separator. The cell pellet was re-suspended in 350µl of buffer RLT (Qiagen), and RNA extracted with RNeasy Plus Mini Kits (Qiagen, 74134). After RNA quality control was confirmed with RIN scores >7.9, cDNA library preparation was performed using a polyA-selected library preparation kit by the Vanderbilt Technologies for Advanced Genomics (VANTAGE) core facility. Multiplexed sequencing was performed on an Illumina sequencer, with 50 BP single ended sequence reads at 30M reads/sample. Demultiplexed FASTQ RNA Seq reads, and alignment were performed as outlined in S. Methods. Gene set enrichment analyses (GSEA) were performed using the clusterProlifer R package [2]. Enrichment score was calculated by comparing the log fold change in each gene in the gene set with all other expressed genes. This represented as the normalized to the gene set size for each gene set to generate normalized enrichment scores (NES). GSEA was performed for Gene Ontology (GO) classification from the MSigDB collections [3,4], as well as validated macrophage and RA target gene sets from published data [5-7], as described in the text.

Validation of the human AKI RNA sequencing dataset. Differentially expressed gene lists comparing control and AKI kidney samples evaluated by bulk and snRNA sequencing for different renal cells data were obtained from S. Table S2 in Hinze et al. Genome Medicine 2022.[1] RNA and snRNA samples were obtained from cadaveric renal biopsies obtained 60-120 minutes after cessation of circulation in 8 patients who died in a clinical care setting with pneumonia associated with severe sepsis-associated AKI (KDIGO 2 and 3) diagnosed within 5 days of sampling . Three age matched control samples were obtained from tumor adjacent normal renal tissue obtained at nephrectomy, and one control for validation of cadaveric donor renal biopsies obtained from timed post mortem biopsies (15, 60 and 120 minutes after circulatory

arrest) in a younger patient with brain death. Principal component analysis (PCA) showed clear separation of control and AKI samples, and control samples obtained after 15, 60 and 120 minutes of circulatory arrest clustered closely with nephrectomy specimens in the two major dimensions (Dm1 and Dm2, accounting for 38 and 13% of variance, respectively). snRNA seq analysis demonstrated very similar distributions of the 12 identified renal cell types between all control and AKI samples. Note that PTECs and TAL cells accounted for ~60% of the cells identified from snRNA seq analysis, with lower %'s of CD and leukocyte populations (~10% and 2% respectively).

Mouse RNA sequencing alignment and analysis. Demultiplexed FASTQ RNA Seq reads were aligned using STAR 2.5.0a, controlling for unannotated, non-canonical and rare junctions. Mouse genome assembly, GRCm38, and corresponding transcript annotations, downloaded from Ensembl, were used for alignment. The htseq-count function from HTSeq 0.6.1 was used in the intersection, non-empty mode to count the aligned reads. The edgeR and limma packages were used for differential expression analysis between the two conditions. [8] [9] Genes with log fold change $\geq 1$ or $\leq -1$ and adjusted p-value $< 0.05$ are considered as differentially expressed.

RNA extraction and quantitative RT-PCR. RNA purification from tissues and cultured cells was performed using RNeasy Mini Kits, according to the manufacturer's instructions (Qiagen, 74106). Frozen tissue was homogenized in the RNeasy lysis buffer mixed with ceramic beads (Lysing Matrix A tubes, MP Biomedicals 6910050) using the Mini Bead Beater (BioSpec Products Inc, 607). RNA was quantified using a Nanodrop Spectrophotometer, and cDNA synthesized from 1$\mu$g RNA with iScript cDNA synthesis kits (Bio-Rad 1708891). Quantitative PCR was performed using iQ SYBR Green super mix (Bio-Rad) using a Bio-Rad CFX96 real time PCR system. mRNA expression was normalized to *Gapdh* mRNA using CFX Maestro software, results reported as normalized expression ($\Delta\Delta$Ct). Primers pairs were downloaded directly from PrimerBank, or designed using the National Center for Biotechnology Information Primer Designing tool, as described [10]. PrimerBlast was used to confirm that primers spanned exon junctions. Primer sequences are listed S. Table 5D.

Mouse mutant lines. Note: *RARE-hsp68-LacZ* were also backcrossed for 9 generations onto a BALB/c background, but *RARE-LacZ* expression was no longer detectable in the kidney after rhabdo-AKI, so this background strain was

discontinued. To select for homozygous *R26R-DN RAR* mice, we performed copy number variant (CNV) genotyping. For this, DNA was extracted using DNeasy Blood and Tissue Mini Kits, per the manufacturer's instructions (Qiagen, 69504). DNA was quantified using a Nanodrop Spectrophotometer, and quantitative PCR performed in triplicate using 10ng of sample DNA with iQ SYBR Green super mix (Bio-Rad) using a Bio-Rad CFX96 real time PCR system. Known wild type, heterozygous and homozygous *R26R DN RAR* DNA samples (established from breeding) were included in each experiment as controls. DNA copy number was determined with primers for *Gapdh* as the reference genomic DNA control using CFX Maestro software: wild type mice have *R26R DN RAR* copy numbers close to 0, heterozygous mice between 0.4 and 0.6, and homozygous mice 0.8 to 1.2. Primer sequences used for CNV genotyping are listed in S. Table 5A.

<u>AKI models</u>. Mice were maintained on a 12:12-h light-dark cycle with free access to standard food and water. Euthanasia was performed by cervical dislocation after anesthesia with inhaled isoflurane at the end of each experiment, or at humane end points. Blood was collected for the determination of serum BUN, and kidneys harvested analysis at the indicated time points. For all injury models, body weight was monitored throughout the study. To induce Rhabdo-AKI, wild type BALB/c mice were given intramuscular (IM) 50% glycerol (Invitrogen, Carlsbad, CA) in sterile water after water deprivation for 18hrs prior to injection, as described [11]. Different doses of glycerol were first determined that would induce moderately severe but survivable AKI in the different mouse strains to induce survivable injury: 6ml/kg glycerol; *RARE-LacZ* mice, 5.5ml/kg glycerol; and PTEC DN RAR mice 5.8ml/kg glycerol. Glycerol was injected into anterior thigh muscles while mice were anesthetized with a ketamine-xylazine mixture (120–150 mg/kg ketamine and 12–15 mg/kg xylazine) IP, with the glycerol dose split equally between the two hindlimbs. Pain relief with Buprenorphine was provided for 48 h starting immediately after intramuscular injections. Mice were also given 0.5ml of subcutaneous (SC) sterile 0.9% saline immediately after and 24 h after intramuscular injections. For bilateral IRI-AKI, 10–12-week-old male PTEC DN RAR mice were anaesthetized with IP ketamine-xylazine mixture and placed prone on a surface heated by a circulating water bath at 38°C. The kidneys were exteriorized via a dorsal incision, and after dissection of the renal pedicle from perinephric fat, placed back into the retroperitoneal space for 8 minutes to allow the kidneys to return to body temperature. 200-240gm pressure clamps (Roboz RS-5459) clamps were then placed on both renal pedicles staggered 1-2 minutes apart, and kidneys returned to the retroperitoneal space, and the mice was covered with a dry gauze pad and foil blanket. After

28 minutes, kidneys were exteriorized, the clamps removed, and kidney observed over 60 seconds for return of pink coloration before returning them to the retroperitoneal space and closing the wound in layers. If kidneys did not fully re-perfuse or there were other technical problems with the surgery, mice were excluded from analyses. 0.5 ml of 0.9% saline was given SC immediately after and the morning after surgery. Pain relief was provided with buprenorphine for 48hrs. All surgeries were started in the morning and completed by noon. For unilateral IRI-AKI with delayed nephrectomy, mice were anaesthetized with a ketamine-xylazine mixture, the left kidney exteriorized, the renal pedicle clamped and then removed after a defined time period . Studies were performed in two separate experiments with 34- and 36-minutes renal pedicle clamp times with no differences in survival or renal function between studies. For the nephrectomy surgery, mice were anesthetized using inhaled isoflurane, the right kidney exteriorized, the renal pedicle was tied off with silk suture, and the kidney removed. Pain relief was provided with SC buprenorphine for 48 h after surgery. Surgeries were started in the morning and completed by noon, and 0.5 ml of SC 0.9% saline was given immediately after and the morning after IRI surgery.

Assessment of renal function. Blood was collected by submandibular vein or cardiac puncture into lithium-heparin-coated microcuvette tubes. Plasma was collected for BUN and measured in duplicate, according to the manufacturer's instructions (Infinity Urea, Thermo Scientific, Waltham, MA). Transdermal glomerular filtration rates (tGFR) was performed in conscious mice, as described [12]. The FITC-sinistrin half-life was calculated using a three-compartment model with linear fit using MPD Studio software (MediBeacon, Mannheim, Germany). The FITC sinistrin half-life was converted to tGFR (in $\mu$l/min) with correction for mouse body weight, as described [12].

Tissue harvesting, beta Galactosidase, and immunofluorescence staining. Mice underwent terminal cardiac perfusion under anesthesia, initially with 0.9% saline solution and right kidney clamped and removed. 2-3mm transverse blocks were cut transversely through the central cortex and medulla and allocated for formalin fixation and paraffin embedding (FFPE) after immersion in 10% buffered formalin (BF, Fisher brand) for 4 hours, or blocks were snap frozen in liquid nitrogen for subsequent RNA extraction. Mice then continued with cardiac perfusion with 10% BF and the left kidney removed and fixed in 10% BF for another hour. After cutting transverse blocks and washing, samples were placed in 30% sucrose in phosphate buffered saline (PBS) overnight, mounted in Tissue-Tek OCT (Sakura), and stored at -80C for

subsequent immunofluorescence or b-Galactosidase staining assays. Beta Galactosidase staining, and antibody co-labeling was performed on FFF sections, as described [13]. Blocking and antibody incubation steps were performed using the universal blocking reagent (Biogenex, Tremont, CA). For co-labeling studies after b-Galactosidase staining, after incubation with X-Gal substrate, sections were fixed in methanol, before antigen retrieval, blocking steps and incubation with primary and secondary antibodies, and/or biotinylated lectins. TUNEL staining was performed using TMR-Red in situ cell death detection kit (Roche, 12156792910) according to the manufacturer's instructions. Briefly, after FFPE slides were de-paraffinized, sections were incubated with Proteinase K (20µg/ml) for 10 min at 37 °C. Slides were then incubated with TUNEL reaction mixture (5µl of Enzyme solution, 45µl of Label solution) at 37 °C for 1 hour, followed by washing in PBS.

Image analysis. For quantification of IF and b-galactosidase-stained images, digital images were scanned using Zeiss AxioScan Z1 slide scanner with EGFP, DAPI, DsRed, and AF 647 filters on (Carl Zeiss Microscopy GmbH, Oberkochen, Germany, 10X), and digital images downloaded into QuPath (version 0.4.3) to generate color overlays. For b-Gal/ IF staining overlays, the bright field blue X-Gal color change was pseudo colored in white and overlaid onto digitally acquired fluorescence images. For quantification, kidney regions (cortex, outer stripe of the outer medulla (OSOM), inner stripe of the outer medulla (ISOM) and inner medulla (IM)) were identified on whole kidney scanned images using established landmarks depending on the experiment (e.g.: juxta medullary glomeruli to demarcate the cortex/OSOM junction, LTL staining the OSOM/ISOM junction; and THP-1 staining to demarcate the ISOM/IM junction). Images were then quantified either as surface area stained in the indicated regions, or the ratio of cells staining with the indicated markers using DAPI staining to quantify individual cell nuclei, as indicated in the figure legends, using QuPath. Cell numbers and cell types (e.g.: $LTL^+$, and $LacZ^+$ cells) in different kidney areas (i.e.: cortex, OSOM, or ISOM), were identified based on average values of fluorescence staining using machine learning and thresholding detection using QuPath. Briefly, the pixel classifier was trained with a minimum of 30 training regions for the cell (DAPI stained nuclei) and cell type (e.g.: $LTL^+$ tubules), and identified as areas of fluorescence above background levels (nucleus threshold, segmentation parameters, cell expansion, smoothed features). Once the machine learning classifier was created, $LTL^+$ (or other marker) were detected in the entire tissue section. Positive cell detection was then used to

identify the labeled proteins (e.g.: LacZ/ Sox9) by thresholding, and these determined in cell types (e.g.: $LTL^+$). Specific protein expression localization with respect to the $LTL^+$ tubules was analyzed.

Histological scoring. Renal tubular injury scores and fibrosis/collagen deposition were determined on FFPE sections after staining with Periodic Acid Schiff (PAS) and Sirius red (SR), respectively, by blinded observers, as described [10,11]. H.Y. evaluated PAS-stained sections to determine tubular injury scores (TIS), as described [11]. R.D. evaluated SR staining, using an Olympus BX-41 microscope equipped with a polarized light filter, using ImageJ to quantify birefringent SR-stained collagen fibrils areas/total surface areas from digitally captured images, as described [10].

Isolation and characterization of cultured PTECs. Primary mouse PTECs were isolated, as described [14]. Briefly, the kidney cortex was minced into small pieces, digested with collagenase (3 mg/mL, Worthington, S3N6800)/Dispase (1 mg/mL, Gibco, 17105-041)/DNAase (0.1 mg/mL, Sigma-Aldrich, 11284932001) in PBS, and passed through a 40µm strainer (Thermo Fisher Scientific, 22363547). PTECs labeled with biotinylated LTL (Vector Laboratories, B-1325-2) were separated on anti-biotin microbeads (Miltenyi Biotec, 130-090-485) and seeded on collagen 1-coated dishes (30µg/mL) and grown in complete PTEC media comprising DMEM/F12 media supplemented with 50 ng/mL hydrocortisone, 5µg/mL insulin/transferrin/selenium, 6.5 ng/mL triiodothyronine, murine EGF (20 ng/mL, Peprotech, 315-09) penicillin/ streptomycin, and 0.5% BSA (Sigma-Aldrich, A3059). Cells were used between passages 2 and 3. For IF staining, cells were plated uniformly at a density of $1 \times 10^5$cells/well in an 8-well chamber slide (ThermoFisher, 154453 or iBidi, 80826). Cells were washed after reaching confluence, fixed with 5% Formalin and permeabilized with 0.1% Triton X-100 in PBS. After blocking with 2.5% BSA, cells were incubated with primary antibodies overnight at 4°C, and incubated with secondary antibodies for 1 hour. Primary and secondary antibodies used to characterize primary PTECs are summarized in S.Table 5B/C. To evaluate cell growth, P2 PTECs were seeded in triplicate onto a 96-well plate at a density of $7.5 \times 10^3$ cells per well. Wells were coated with collagen 1, and cells grown in complete PTECs media (as outlined above). The plate was placed in a 37°C, 5% $CO_2$ incubator. Cells were counted after 6 hours (t=0), 24 and 72 hours.  For cell counts, wells were washed in PBS, fixed with 100% methanol, stained with DAPI, and nuclear counts determined from digital images acquired from two randomly selected 10X objective fields per well (6 fields/mouse) at each of the indicated time points.

Seahorse assays. Extracellular acidification rates (ECAR, XF Glycolytic Stress Test) and oxygen consumption rates (OCR, XF Cell Mito Stress Test) were performed with a Seahorse XF24 Extracellular Flux Analyzer and test kits (Agilent Technologies, Inc., Santa Clara, CA, USA). Passage 2 PTECs were seeded overnight in quintuplicate at a density of $5 \times 10^4$ cells per well on a Seahorse cell culture plate in PTEC complete media. For ECAR assays, PTECs were incubated in Seahorse assay medium (Agilent Technologies) supplemented with 1 mM L-glutamine in a 37°C incubator without CO2 for 45 min prior to starting the assay. Glucose, oligomycin and 2-DG were injected, according to the manufacturer's instructions. For OCR assays, cells were washed and incubated in Seahorse assay medium supplemented with 1 mM sodium pyruvate, 2 mM L-glutamine, and 25 mM Glucose in a 37°C incubator without CO2 for 45 min. Oligomycin FCCP and rotenone/antimycin were injected per the manufacturer's instructions. The ECAR (mpH/min) and OCR (pMoles O2/min) was measured in real time, and glycolytic and mitochondrial parameters calculated using Wave software (Agilent). After the assays, cells were fixed with 100% Methanol and stained with DAPI solution. Values were normalized for count of DAPI stained nuclei in each well.

**Flow cytometry.** Kidneys were homogenized to single cell suspensions, as outlined above, and residual RBCs lysed with RBC lysis buffer (eBioscience) before proceeding with staining. For dead cells exclusion, Live/Dead Fixable Dead Cell Stain was added to cells. Using anti-mouse CD16/32 to block nonspecific Fc binding, cells were then incubated with cell surface antibodies in FACS buffer for 30 minutes on ice in the dark. Antibodies, dilutions, and fluorophores used are summarized in S.Table 5E. For intracellular (IC) antibody (CD206) staining, cells were fixed and permeabilized with fixation and permeabilization buffer (eBioscience) subsequently, then incubated with IC antibody the same as antibody above. For EdU detection, 1mg/mouse EdU (Invitrogen, C10424) was administrated IP 2- or 24-hours before sacrifice. Following cell surface antibody staining and cell fixation and permeabilization, cells were resuspended in Click-iT Plus reaction cocktail solution and incubated for 30min on ice. Additionally, fluorescence-minus-one (FMO) controls were prepared for each cell marker. Data were collected on a LSR II (BD, San Jose, USA) equipped with five aligned 355, 405, 488,532 and 633 nm lasers. The data analysis including sequential gating based on FMO control was performed by using BD FACS diva software. All antibodies and fluorophores used are listed in S. Table 5E.

Supplemental Tables

**S. Table 1. RAR target genes lists used for GSEA of human RNA Seq data. A,** Validated RAR target genes from Balmer et al. 2002 [7]. **B,** RA signaling regulators previously shown to be upregulated by RAR signaling after IRI-AKI [13].

**S. Table 2. GSEA analysis of RAR target genes in human SA-AKI RNA Seq databases from Hinze et al. 2022[15].** Tab labels indicate GSEA analysis on bulk RNA Seq data for the whole kidney, PTECs, leukocytes, TAL, collecting duct principal cells, interstitial cells and endothelial cells.

**S. Table 3. GSEA for the GO in mouse kidney CD11B bulk RNA Seq data from PTEC DN RAR mice after IRI-AKI.** Tab labels indicate summary of the analysis, GO term upregulated and downregulated gene sets along with core enrichment gene IDs driving significant associations with the bulk RNA Seq data.

**S. Table 4. M1 and M2 activated macrophage gene lists used for GSEA analysis CD11B bulk RNA Seq data.** Validated macrophage marked gene set obtained from Jablonski et al. 2015 [5]. Tab labels indicate individual gene sets.

**S. Table 5. Primers and antibodies used for genotyping, QRT-PCR, immunofluorescence, and flow cytometry studies. A,** Genotyping primer pairs. **B,** Primary antibodies and lectins used for immunofluorescence studies. **C,** Secondary antibodies and fluorophores used from immunofluorescence studies. **D,** Primers used for QRT-PCR studies. **E,** Antibodies and fluorophores used for flow cytometry studies.

References


1. Hinze C, Karaiskos N, Boltengagen A, Walentin K, Redo K, Himmerkus N, Bleich M, Potter SS, Potter AS, Eckardt KU, et al. Kidney Single-cell Transcriptomes Predict Spatial Corticomedullary Gene Expression and Tissue Osmolality Gradients. *J Am Soc Nephrol*. 2021;32:291-306. doi: 10.1681/ASN.2020070930
2. Yu G, Wang LG, Han Y, He QY. clusterProfiler: an R package for comparing biological themes among gene clusters. *OMICS*. 2012;16:284-287. doi: 10.1089/omi.2011.0118
3. Ashburner M, Ball CA, Blake JA, Botstein D, Butler H, Cherry JM, Davis AP, Dolinski K, Dwight SS, Eppig JT, et al. Gene ontology: tool for the unification of biology. The Gene Ontology Consortium. *Nat Genet*. 2000;25:25-29. doi: 10.1038/75556
4. Gene Ontology C. The Gene Ontology resource: enriching a GOld mine. *Nucleic Acids Res*. 2021;49:D325-D334. doi: 10.1093/nar/gkaa1113
5. Jablonski KA, Amici SA, Webb LM, Ruiz-Rosado Jde D, Popovich PG, Partida-Sanchez S, Guerau-de-Arellano M. Novel Markers to Delineate Murine M1 and M2 Macrophages. *PLoS One*. 2015;10:e0145342. doi: 10.1371/journal.pone.0145342
6. Lever JM, Hull TD, Boddu R, Pepin ME, Black LM, Adedoyin OO, Yang Z, Traylor AM, Jiang Y, Li Z, et al. Resident macrophages reprogram toward a developmental state after acute kidney injury. *JCI Insight*. 2019;4. doi: 10.1172/jci.insight.125503
7. Balmer JE, Blomhoff R. Gene expression regulation by retinoic acid. *J Lipid Res*. 2002;43:1773-1808. doi: 10.1194/jlr.r100015-jlr200
8. Robinson MD, McCarthy DJ, Smyth GK. edgeR: a Bioconductor package for differential expression analysis of digital gene expression data. *Bioinformatics*. 2010;26:139-140. doi: 10.1093/bioinformatics/btp616
9. Ritchie ME, Phipson B, Wu D, Hu Y, Law CW, Shi W, Smyth GK. limma powers differential expression analyses for RNA-sequencing and microarray studies. *Nucleic Acids Res*. 2015;43:e47. doi: 10.1093/nar/gkv007
10. Scarfe L, Menshikh A, Newton E, Zhu Y, Delgado R, Finney C, de Caestecker MP. Long-term outcomes in mouse models of ischemia-reperfusion-induced acute kidney injury. *Am J Physiol Renal Physiol*. 2019;317:F1068-F1080. doi: 10.1152/ajprenal.00305.2019
11. Menshikh A, Scarfe L, Delgado R, Finney C, Zhu Y, Yang H, de Caestecker MP. Capillary rarefaction is more closely associated with CKD progression after cisplatin, rhabdomyolysis, and ischemia-reperfusion-induced AKI than renal fibrosis. *Am J Physiol Renal Physiol*. 2019;317:F1383-F1397. doi: 10.1152/ajprenal.00366.2019
12. Scarfe L, Schock-Kusch D, Ressel L, Friedemann J, Shulhevich Y, Murray P, Wilm B, de Caestecker M. Transdermal Measurement of Glomerular Filtration Rate in Mice. *J Vis Exp*. 2018. doi: 10.3791/58520
13. Chiba T, Skrypnyk NI, Skvarca LB, Penchev R, Zhang KX, Rochon ER, Fall JL, Paueksakon P, Yang H, Alford CE, et al. Retinoic Acid Signaling Coordinates Macrophage-Dependent Injury and Repair after AKI. *J Am Soc Nephrol*. 2016;27:495-508. doi: 10.1681/ASN.2014111108
14. Borza CM, Bolas G, Bock F, Zhang X, Akabogu FC, Zhang MZ, de Caestecker M, Yang M, Yang H, Lee E, et al. DDR1 contributes to kidney inflammation and fibrosis by promoting the phosphorylation of BCR and STAT3. *JCI Insight*. 2022;7. doi: 10.1172/jci.insight.150887
15. Hinze C, Kocks C, Leiz J, Karaiskos N, Boltengagen A, Cao S, Skopnik CM, Klocke J, Hardenberg JH, Stockmann H, et al. Single-cell transcriptomics reveals common epithelial response patterns in human acute kidney injury. *Genome Med*. 2022;14:103. doi: 10.1186/s13073-022-01108-9
